# Overexpression of the Novel Arabidopsis Gene *At5g02890* Alters Inflorescence Stem Wax Composition and Affects Phytohormone Homeostasis

**DOI:** 10.3389/fpls.2017.00068

**Published:** 2017-01-26

**Authors:** Liping Xu, Viktoria Zeisler, Lukas Schreiber, Jie Gao, Kaining Hu, Jing Wen, Bin Yi, Jinxiong Shen, Chaozhi Ma, Jinxing Tu, Tingdong Fu

**Affiliations:** ^1^National Key Laboratory of Crop Genetic Improvement, National Center of Rapeseed Improvement, Huazhong Agricultural UniversityWuhan, China; ^2^Institute of Cellular and Molecular Botany, University of BonnBonn, Germany

**Keywords:** *Arabidopsis thaliana*, *At5g02890*, *Brassica napus*, cuticular wax, phytohormones, VLCFAs

## Abstract

The cuticle is composed of cutin and cuticular wax. It covers the surfaces of land plants and protects them against environmental damage. *At5g02890* encodes a novel protein in *Arabidopsis thaliana*. In the current study, protein sequence analysis showed that At5g02890 is highly conserved in the *Brassicaceae*. Arabidopsis lines overexpressing *At5g02890* (OE-At5g02890 lines) and an *At5g02890* orthologous gene from *Brassica napus* (OE-Bn1 lines) exhibited glossy stems. Chemical analysis revealed that overexpression of *At5g02890* caused significant reductions in the levels of wax components longer than 28 carbons (C28) in inflorescence stems, whereas the levels of wax molecules of chain length C28 or shorter were significantly increased. Transcriptome analysis indicated that nine of 11 cuticular wax synthesis-related genes with different expression levels in OE-At5g02890 plants are involved in very-long-chain fatty acid (VLCFA) elongation. At5g02890 is localized to the endoplasmic reticulum (ER), which is consistent with its function in cuticular wax biosynthesis. These results demonstrate that the overexpression of *At5g02890* alters cuticular wax composition by partially blocking VLCFA elongation of C28 and higher. In addition, detailed analysis of differentially expressed genes associated with plant hormones and endogenous phytohormone levels in wild-type and OE-At5g02890 plants indicated that abscisic acid (ABA), jasmonic acid (JA), and jasmonoyl-isoleucine (JA-Ile) biosynthesis, as well as polar auxin transport, were also affected by overexpression of *At5g02890*. Taken together, these findings indicate that overexpression of *At5g02890* affects both cuticular wax biosynthesis and phytohormone homeostasis in Arabidopsis.

## Introduction

The cuticle is an extracellular lipidic layer on the vascular plant surface that primarily consists of cutin and cuticular wax. The primarily functions of the cuticle are to restrict non-stomatal water loss and to limit the deposition of dust onto the plant surface (Barthlott and Neinhuis, 1997; Riederer and Schreiber, 2001). As the outermost layer of the plant, the cuticle has also evolved secondary functions to protect the plant against various biotic and abiotic stresses, such as pests, pathogens, ultraviolet radiation, toxic gas, mechanical rupture, and drought stress (Kunst and Samuels, 2003; Pfündel et al., 2006; Leide et al., 2007; Reina-Pinto and Yephremov, 2009; Lü et al., 2012; Yeats and Rose, 2013). Furthermore, the cuticle is an active component of plant development, which prevents epidermal fusion by establishing normal organ boundaries (Bellec et al., 2002; Yeats and Rose, 2013) and is also involved in phytohormone homeostasis (Roudier et al., 2010; Wang et al., 2011; Lü et al., 2012; Nobusawa et al., 2013).

Cutin, as a polyester structure, is primarily composed of C16 and C18 hydroxy and epoxy fatty acid monomers, which are cross-linked by ester bonds, besides that, cutin also contains glycerol and small amounts of phenolic compounds (Heredia, 2003; Pollard et al., 2008; Kunst and Samuels, 2009; Beisson et al., 2012). Cuticular wax consists of two fractions: intracuticular wax, which is embedded in the cutin matrix, and epicuticular wax, which is present on the outer cutin surface. Cuticular wax generally comprises a complex mixture of very-long-chain fatty acids (VLCFAs), aldehydes, alkanes, ketones, alcohols, with chain lengths typically varying from C20 to C34, and esters with chain lengths varying from C38 to C52 (Jenks and Ashworth, 1999; Jetter et al., 2006; Samuels et al., 2008).

The major steps in cuticular wax biosynthesis have been defined. This process begins with *de novo* saturated C16 or C18 fatty acid biosynthesis in the plastid. The C16 and C18 fatty acids are then catalyzed by elongase complexes in the endoplasmic reticulum (ER), which generates VLCFA-CoAs ranging from C20 to C34. After being synthesized, VLCFA-CoAs are converted into free VLCFAs or other wax precursors through two distinct pathways: the acyl-reduction (alcohol-forming) pathway, which produces 17–18% of the total wax content, including even-chain primary alcohols and wax esters (Kunst and Samuels, 2003; Rowland et al., 2006; Li et al., 2008); and the decarbonylation (alkane-forming) pathway, which form compounds accounting for more than 80% of the total wax content, including aldehydes and odd-chain alkanes, secondary alcohols and ketones (Schneider-Belhaddad and Kolattukudy, 2000; Greer et al., 2007; Bernard et al., 2012). VLCFAs are synthesized by a multi-enzyme fatty acid elongase (FAE) complex (Millar et al., 1999; Fiebig et al., 2000; Kunst and Samuels, 2009). The FAE complex is composed of four enzymes, including β-ketoacyl-CoA synthase (KCS), β-ketoacyl-CoA reductase (KCR), β-hydroxyacyl-CoA dehydratase (HCD), and enoyl-CoA reductase (ECR). This complex, which is localized to the ER, catalyzes four successive reactions that generate a fatty acyl chain extended by two carbon units (Lee and Suh, 2013; Li-Beisson et al., 2013). The first elongation step, which is catalyzed by KCSs, is the rate-limiting step in the pathway (Millar and Kunst, 1997; Haslam and Kunst, 2013). To date, 21 putative KCSs have been annotated in Arabidopsis, and some KCSs have successfully been characterized in detail (James et al., 1995; Millar et al., 1999; Fiebig et al., 2000; Dunn et al., 2004; Joubès et al., 2008; Bernard and Joubès, 2013). In Arabidopsis, *CER6* plays a major role in the production of fatty acids with 26 or 28 carbon atoms (Millar et al., 1999; Fiebig et al., 2000; Tresch et al., 2012), *CER2* is involved in the synthesis of C30 VLCFAs in conjunction with *CER6* or *CER60* (Haslam et al., 2012, 2015), *CER2-LIKE1* (*CER26*) is specifically involved in elongating VLCFAs to create molecules with chain lengths greater than C30 (Pascal et al., 2013; Haslam et al., 2015). *CER60* is the only KCS with demonstrated C28–C30 elongation activity, but it only produces trace amounts of C30 VLCFAs when expressed in yeast (Trenkamp et al., 2004). Despite numerous efforts, our knowledge of the specific mechanism of VLCFA elongation from C28 to C30 remains incomplete.

Overexpression of *CER26* displays a glossy stems phenotype and leads to the production of cuticular wax composition with more than 30 carbon atoms in stems (Pascal et al., 2013). At5g02890 encodes a putative BAHD acyltransferase with increased expression levels in *CER26*-overexpressing (OE-CER26) plants. The BAHD acyltransferase family was named from the first letter of its first four characterized enzymes: benzylalcohol O-acetyltransferase (BEAT), anthocyanin O-hydroxycinnamoyltransferase (AHCT), anthranilate N-hydroxycinnamoyl/benzoyltransferase (HCBT), and deacetylvindoline 4-O-acetyltransferase (DAT; St-Pierre and De Luca, 2000). The three genes involved in VLCFA elongation from C28 to C34, *CER2, CER26*, and *CER26-LIKE*, are all putative BAHD acyltransferase. Therefore, we hypothesized that At5g02890 might also influences cuticular wax biosynthesis.

In the current study, we characterized the expression patterns of *At5g02890* and found that At5g02890 is localized to the ER. Arabidopsis plants overexpressing *At5g02890* and the *At5g02890* orthologous gene from *Brassica napus* had glossy stems. Subsequently, through cuticular wax composition analysis, transcriptome analysis, and determination of endogenous phytohormones levels in wild-type and OE-At5g02890 plants, we aimed to answer the following questions: (1) Does *At5g02890* influence cuticular wax biosynthesis in OE-At5g02890 plants? (2) How might *At5g02890* affect cuticular wax biosynthesis in OE-At5g02890 plants? (3) Does overexpressing *At5g02890* influence other metabolic pathways in the plant?

## Materials and methods

### Plant materials and growth conditions

*Arabidopsis thaliana* Col-0 was used as the wild-type control in all experiments. The overexpression and RNAi lines were constructed via transgenic technology. The seeds were vernalized for 3–4 days at 4°C and germinated in soil. For all experiments, the Arabidopsis plants were grown in controlled greenhouse conditions under long-day conditions (16-h-light/8-h-dark cycle) at 21–23°C with 30–60% humidity.

### Protein sequences alignment and phylogenetic analysis

The full-length amino acid sequence of At5g02890 and the closest 35 sequences identified via BLASTP search were aligned using the MUSCLE tool (http://www.ebi.ac.uk/Tools/services/web_muscle/), and a phylogenetic tree was constructed from the aligned sequences using MEGA5.2 (Windows) (www.megasoftware.net/; Tamura et al., 2011).

### Cloning and generation of transgenic plants

The coding sequences of *At5g02890* and *At5g02890* orthologous gene were amplified from Arabidopsis and *B. napus* cDNA using the primers shown in Table S1. The PCR products were cloned into the pMD18-T vector to produce intermediate vectors, which were then sequenced. The intermediate vectors and the PBI12S binary vector were digested with the same restriction endonucleases. The digested coding sequences were transferred into the digested PBI12S, followed by ligation of the digestion products. The vectors expressed the insert under the control of the 35S promoter. Finally, the overexpression constructs were introduced into Arabidopsis by *Agrobacterium tumefaciens*-mediated transformation using the floral dip method (Clough and Bent, 1998). Transgenic lines were identified by PCR prior to subsequent analysis.

### GUS assay

A DNA fragment containing 1481 bp of the sequence immediately upstream of the ATG start codon of *At5g02890* was PCR-amplified from wild-type Arabidopsis plant genomic DNA using the At5g02890pro-F/At5g02890pro-R primers (Table S1) and cloned into the pCAMBIA2300 binary vector. The Pro_*At5g02890*_-GUS construct was introduced into wild-type Arabidopsis plants by *Agrobacterium*-mediated transformation. The GUS activity was visualized by overnight staining of different tissues from the transgenic lines with 5-bromo-4-chloro-3-indolyl-β-D-glucuronide (X-Gluc) solution (Willemsen et al., 1998). Subsequently, the tissues were cleared with 75% (v/v) ethanol and visualized under a stereo light microscope. The GUS staining solution was composed of 50 mM sodium phosphate (pH 7.0), 0.5 mM potassium ferricyanide, 0.5 mM potassium ferrocyanide, 0.1% (v/v) Triton X-100, and 0.5 mg/mL X-Gluc.

### RT-PCR and qRT-PCR analyses

Total RNA was extracted from frozen material using TRIzol reagent (Invitrogen, Carlsbad, CA), and first-strand cDNA was synthesized from 1 μg of total RNA using the Toyobo ReverTra Ace RT-PCR system (Toyobo, Japan) according to the manufacturer's instructions. The qRT-PCR was performed using the Bio-Rad CFX96 Real-Time system (Bio-Rad) with SYBR Green Real-Time PCR Master Mix (Toyobo, Japan) according to the manufacturer's instructions. Four biological replicates were performed per experimental line. The data were analyzed using LinReg software as previously described (Ramakers et al., 2003). *ACTIN7* (*At5g09810*) and *ACTIN2* (*At3g18780*) were used as a constitutive control, and all primers used are shown in Table S1.

### Cryo-SEM and TEM analysis

For cryo-SEM analysis, fresh samples from the wild-type and overexpression lines were rapidly frozen in liquid nitrogen, cracked open, and sputtered with a thin gold layer in the Quorum PP3010T (England) Cryo-SEM Transfer system. The coated samples were observed under a Hitachi SU8010 (Japan) scanning electron microscope at 3 kV and a working distance of 8.3 mm.

For TEM analysis, the samples were vacuum-infiltrated and pre-fixed in a solution of 2.5% glutaraldehyde adjusted to pH 7.4 with 0.1 M phosphate buffer. The samples were then fixed in 2% OsO_4_ in the same buffer, dehydrated, and embedded in epoxy resin and SPI-812. Subsequently, ultra-thin sections were produced using a Leica UC6 ultramicrotome and were successively stained with uranyl acetate and lead citrate. The images were visualized and recorded using a Hitachi H-7650 transmission electron microscope at 80 kV connected to a Gatan 832 CCD camera.

### Wax extraction and analysis

The cuticular wax composition of the stems of 6-week-old plants was determined according to Hauke and Schreiber (1998) with the slight modifications described by Lü et al. (2009). Cuticular wax was extracted using chloroform, and 10 μg of tetracosane (C24 alkane, 10 mg/50 mL) was added as an internal standard. The wax extracts were derivatized in 20 μL of N,N-bis(trimethylsilyl)-trifluoroacetamide (BSTFA; Machery-Nagel, Düren, Germany) and 20 μL of dry pyridine (GC-grade, Merck, Darmstadt, Germany) for 40 min at 70°C. Quantitative analysis was performed using gas chromatography and flame ionization detection (Type: 6890N, Agilent Technologies, USA). Qualitative wax analysis was performed using gas chromatography and mass spectrometry (Type: 5973N, Agilent Technologies, USA).

### Subcellular localization

The full-length CDS of *At5g02890* without the stop codon (TGA) was PCR-amplified using the At5g02890SL-F/At5g02890SL-R primers (Table S1) and inserted into the pM999GFP vector. An red fluorescent protein (RFP) fusion with the chaperone-binding protein (BiP) was used as an ER marker protein (Kim et al., 2001). mRFP was used as a cytosol marker protein (Kim et al., 2016). The two fusion constructs were introduced into Arabidopsis protoplasts by PEG/calcium-mediated transformation (Yoo et al., 2007). Fluorescence signals were detected and photographed under a confocal laser microscope.

### High-throughput RNA sequencing and data analysis

Total RNA was isolated from inflorescence stems (15 days after bolting) of the OE-At5g02890 and wild-type lines, including OE-1, OE-2, OE-3, W-1, W-2, and W-3 (three biological replicates per line), using TRIzol reagent (Invitrogen, Carlsbad, CA). The samples were sequenced on the Illumina HiSeq™ 2500 platform. The sequencing data sets for each sample were ~2G in size. The project was submitted to NCBI BioProject with BioProject ID: PRJNA356835. The raw reads were deposited in NCBI SRA (Short Read Archive) with accession number SRP094871. Clean reads were obtained from the raw data by filtering the adaptor sequences and low-quality sequences using SolexaQA (Cox et al., 2010). Gene expression levels were calculated and normalized to FPKM (Fragments per Kilobase of transcript per Million mapped reads). The GO annotations for the unigenes were determined using Blast2GO (Conesa et al., 2005). The DEGs were annotated to KEGG reference pathways using KeggArray software.

### Quantification of endogenous IAA, ABA, JA, and JA-Ile

To determine the endogenous phytohormone levels in the plants, 0.1 g (fresh weight) samples were ground to a fine power in liquid nitrogen and homogenized in 750 μL cold extraction buffer (methanol: water: acetic acid, 80:19:1, v/v/v) supplemented with internal standards. The subsequent extraction procedure was performed according to a previous report (Liu et al., 2012). The extracts were filtered through a 0.22 μm nylon membrane (Nylon 66; Jinteng Experiment Equipment Co., Ltd, Tianjing, China) and dried under the flow of nitrogen gas at room temperature. Finally, the dried powders were dissolved in 200 μL methanol and stored at −70°C until use. Quantification of endogenous IAA, ABA, JA, and JA-Ile was performed according to a previous report (Liu et al., 2012).

## Results

### *At5g02890* encodes a putative transferase with an unknown function

The open reading frame (1062 bp) of *At5g02890* encodes a protein consisting of 353 amino acids, which is annotated as a putative transferase. By transcriptome and quantitative real-time PCR (qRT-PCR) analysis, we found that the expression levels of *At5g02890* were highly increased in OE-CER26 lines (Figure S1). To predict the possible function of At5g02890 in plants, we used the sequence of the full-length At5g02890 protein as a query in a BLASTP search for homologs in the following six public databases: the National Center for Biotechnology Information (NCBI, http://www.ncbi.nlm.nih.gov/), The Arabidopsis Information Resource (TAIR, http://www.arabidopsis.org/), the Brassica Database (http://brassicadb.org/brad/), the *Brassica oleracea* Genomics Database (http://www.ocri-genomics.org/bolbase/index.html), the *B. napus* Genomics Database (http://brassicadb.org/brad/datasets/pub/Genomes/Brassica_napus/), and the Rice Genome Annotation Database (http://rice.plantbiology.msu.edu/). In total, 35 close homologs of At5g02890 were obtained (Table S2). Subsequently, we constructed a neighbor-joining phylogenetic tree with these homologous protein sequences. At5g02890 and its homologs were grouped into two main clades: clade I contains 18 homologs from seven species with levels of sequence identity to At5g02890 ranging from 64 to 88%, and clade II contains 17 homologs from 13 species with levels of sequence identity to At5g02890 ranging from 36 to 48% (Figure S2 and Table S2). Interestingly, none of the proteins have been described in detail, indicating that *At5g02890* encodes a protein of unknown function.

### Expression patterns of *At5g02890* in Arabidopsis

**Figure 1:**
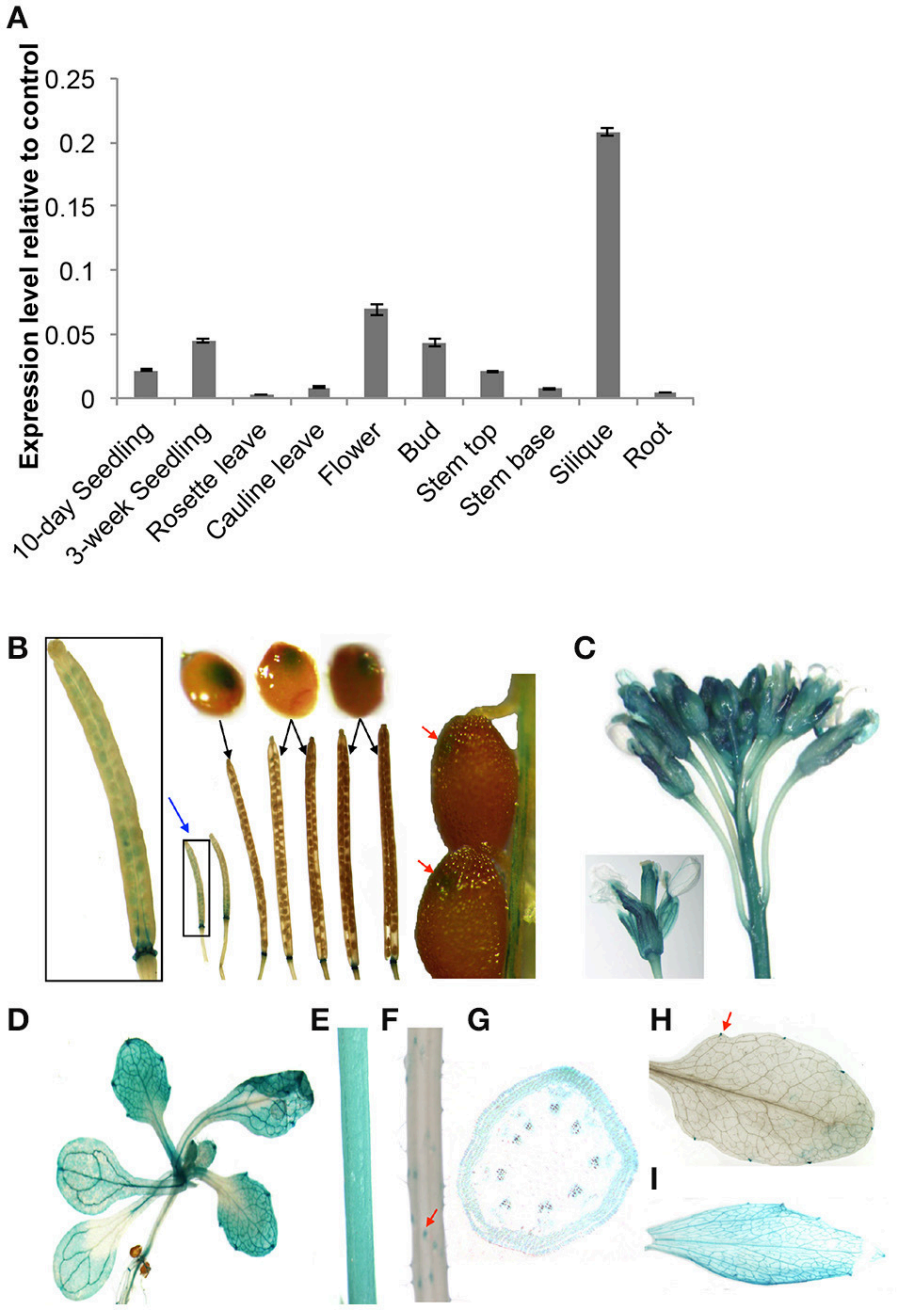
**Expression patterns of *At5g02890* in Arabidopsis. (A)** Analysis of *At5g02890* expression levels in various organs. The gene expression levels were determined by quantitative real-time PCR (qRT-PCR) analysis. The results are presented as relative transcript abundance. The data represent the means ± standard deviation (*SD*) of four biological replicates. **(B–H)** Spatial expression patterns of *At5g02890* in transgenic Arabidopsis plants harboring the *At5g02890* promoter fused to the GUS gene. Promoter activity was visualized through histochemical GUS staining of **(B)** seeds throughout the growth period and abscission zones at the bottom of siliques, **(C)** buds and flowers, **(D)** 10-day-old seedlings, **(E)** stem tops, **(F)** stem bases, **(G)** transverse section of stem top, **(H)** hydathodes of rosette leaves, and **(I)** cauline leaves. Red arrows indicate specific staining sites. Blue arrow indicates magnified image. Black arrows indicate seeds during the corresponding periods of siliques.

We evaluated the spatial and temporal expression patterns of *At5g02890* using qRT-PCR analysis. *At5g02890* transcripts were detected at high levels in siliques, at moderate levels in seedlings, flowers, buds, and stem tops, and at low levels in cauline leaves and stem bases (Figure 1A). This result is consistent with the expression profiling results obtained from the Arabidopsis eFP Browser (http://bar.utoronto.ca/efp/cgi-bin/efpWeb.cgi).

To further investigate the expression patterns of *At5g02890*, we fused a 1.5-kb fragment of the *At5g02890* promoter to the *Escherichia coli* β-glucuronidase (GUS) reporter gene and transformed it into Arabidopsis. Consistent with the qRT-PCR results, GUS signals were detected in seeds throughout the entire growth period (GUS signals were visible by eye during early seed growth but only microscopically as the seeds matured). In addition, GUS signals were detected in the abscission zones at the bottoms of siliques as well as in buds, flowers, seedlings, stem tops, trichomes of stem bases, hydathodes of rosette leaves and cauline leaves (Figures 1B–F,H,I). The GUS signal was observed in the epidermis cells of stem tops, but it was not epidermis-specific (Figure 1G). These results indicate that *At5g02890* is expressed in both vegetative and reproductive organs.

### Overexpression of *At5g02890* and *At5g02890* orthologous genes from *Brassica napus* in Arabidopsis causes glossy stems

**Figure 2:**
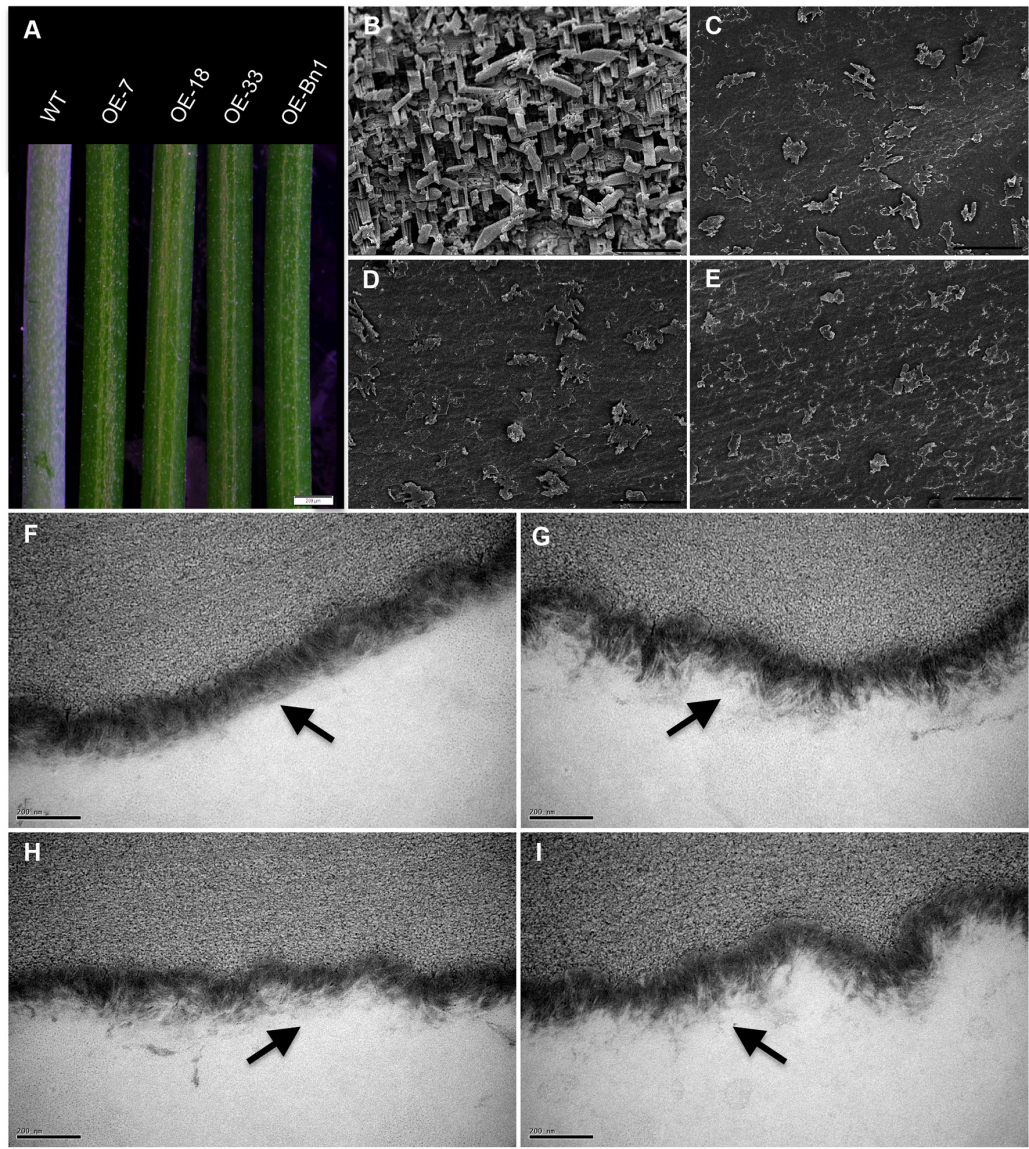
**Phenotypic characterization of OE-At5g02890 and OE-Bn1 plants. (A)** Stems of WT and transgenic lines were photographed under a stereoscopic microscope. **(B–E)** Epicuticular wax crystal formation on Arabidopsis stem surfaces detected by cryogenic-scanning electron microscopy. Scale bars = 5 μm. **(F–I)** Arabidopsis stem epidermal cuticle examined by transmission electron microscopy. The two layers of the cuticle, the outer cuticle proper, and the inner cuticular layer, lie just below the black arrows. Scale bars = 200 nm. Wild type **(B,F)**, OE-7 **(C,G)**, OE-18, **(D,H)**, and OE-Bn1 **(E,I)**. OE-7, OE-18, OE-33 represent different *At5g02890*-overexpressing lines, Bn1 represents *At5g02890* orthologous gene from *Brassica napus*.

**Figure 3:**
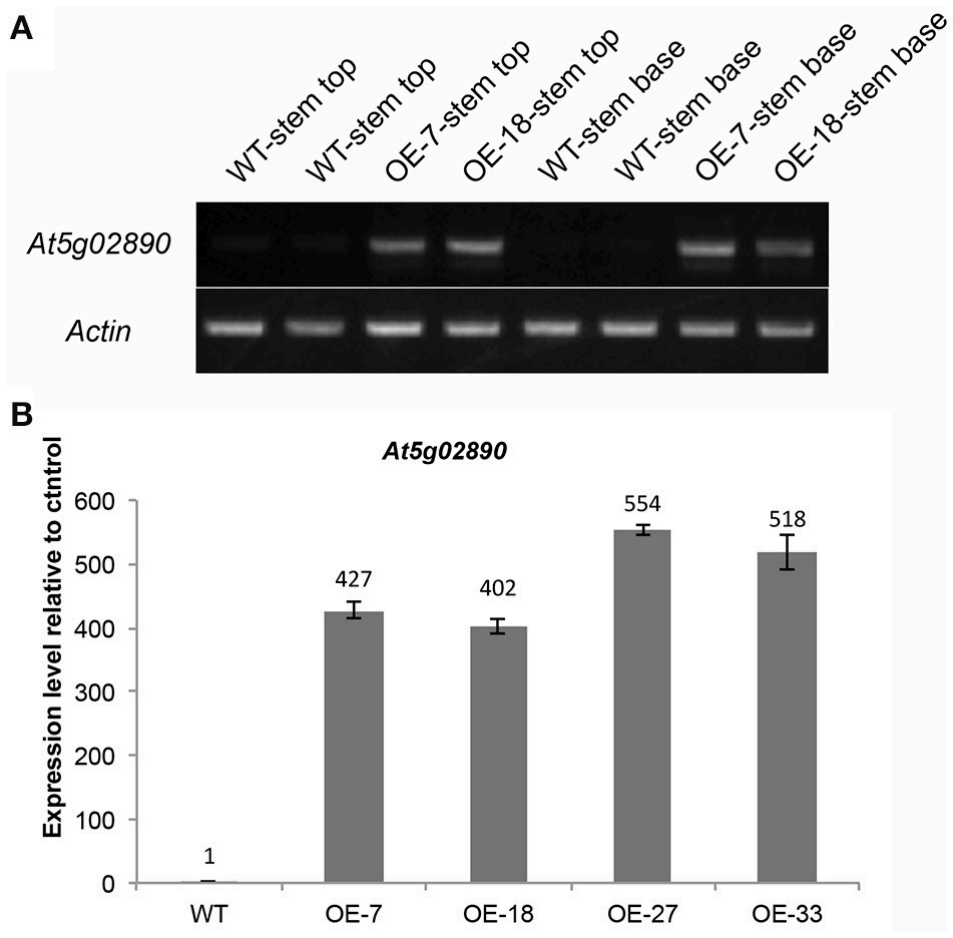
**Expression analysis of *At5g02890* in OE-At5g02890 plants. (A)** Semiquantitative RT-PCR analysis of *At5g02890* transcript levels in stem tops and stem bases of WT and *At5g02890*-overexpressing lines. The *Actin7* gene was used as a constitutively expressed control. **(B)** qRT-PCR analysis of *At5g02890* expression levels in inflorescence stems of WT and *At5g02890*-overexpressing plants. The results are presented as relative transcript abundances. The data represent the means ± *SD* of four biological replicates. OE-7, OE-18, OE-27, and OE-33 represent different *At5g02890*-overexpressing lines.

To investigate the function of At5g02890, we obtained SALK_064174, SALK_064193, and SALK_064194 T-DNA insertion lines from the Arabidopsis Biological Resource Center (http://www.arabidopsis.org). Unfortunately, the later generations of *SALK_064174* and *SALK_064194* exhibited a wild-type genotype without any T-DNA insertion. Only the progeny of *SALK_064193* were homozygous T-DNA insertion plants, but the insertion site was verified after the stop codon and *At5g02890* transcript levels were not reduced compared with wild-type plants (Figures S3A,B). Subsequently, we generated six constructs, including *35S::At5g02890-1(antisense)-intron-At5g02890-1 (sense), 35S::At5g02890-2(antisense)-intron-At5g02890-2(sense), 35S::At5g02890-3(antisense)-intron-At5g02890-3 (sense), At5g02890pro::At5g02890-1(antisense)-intron-At5g02890-1 (sense), At5g02890pro::At5g02890-2 (antisense)-intron- At5g02890-2(sense), and At5g02890pro::At5g02890-3(antisense)-intron-At5g02890-3(sense)* (*At5g02890-1*: 290 bp in the 5′ UTR; *At5g02890-2*: 311 bp in the 3′ UTR; *At5g02890-3*: 327 bp in the coding region), which we used to produce RNA interference lines. Similar to the T-DNA insertion lines, we only obtained five RNA interference plants, none of which showed significantly reduced expression of *At5g02890* (Figure S3C). We also amplified the coding sequence of *At5g02890* and cloned it into the PBI121S binary vector under the control of the *Pro35S* promoter. The resulting construct was introduced into Arabidopsis via *A. tumefaciens*-mediated transformation. Forty-nine lines overexpressing *At5g02890* were obtained, which are referred to as OE-At5g02890 lines. Interestingly, the stems of all OE-At5g02890 plants showed a visibly glossy phenotype (Figure 2A), which is typically associated with wax-deficient mutations and indicates qualitative or quantitative modifications in the production of cuticular wax on the stem surface. Four of the OE-At5g02890 lines, namely OE-7, OE-18, OE-27, and OE-33, were selected randomly for further analysis. To examine the glossy phenotype resulting from *At5g02890* overexpression, we investigated *At5g02890* transcript levels in inflorescence stems from the OE-At5g02890 lines by semiquantitative reverse-transcription PCR (RT-PCR) and qRT-PCR analyses using wild-type plants as a control. The expression level of *At5g02890* was improved by 402 times to 554 times in OE-At5g02890 plants than in wild type (Figures 3A,B). These results indicate that the glossy stem phenotype was caused by the overexpression of *At5g02890*.

Four homologous genes from *B. napus, Bn1, Bn2, Bn3*, and *Bn4*, were also identified and cloned into the PBI121S binary vector under the control of the *Pro35S* promoter (Figure S4). We transformed Arabidopsis with these constructs, obtaining more than 20 transformants per constructs. Interestingly, only overexpression of *Bn1* led to the production of Arabidopsis plants with glossy stems (herein named OE-Bn1; Figure 2A).

### Defective wax deposition and cuticle structure in OE-At5g02890 and OE-Bn1 inflorescence stems

In general, wax crystals and/or cuticle structure defects result in a glossy phenotype. Therefore, we examined the stem surfaces of the transformants via cryogenic-scanning electron microscopy (cryo-SEM) and transmission electron microscopy (TEM) (Jeffree, 2006). The cryo-SEM analysis revealed that epicuticular wax structure in the OE-At5g02890 and OE-Bn1 plants was significantly impaired, with abnormal, reduced numbers of crystals observed in these lines compared with the heavy deposition of wax crystals on the wild-type stem surface (Figures 2B–E). TEM analysis revealed that the cuticles of OE-At5g02890 and OE-Bn1 stems had clearly altered ultrastructure compared to wild type. Wild-type stem cuticles had a typical type-3 cuticle structure (Jeffree, 1996; Lü et al., 2009; Figure 2F), whereas OE-At5g02890 and OE-Bn1 stems had disorganized, thinner cuticles (Figures 2G–I). Electron density signals reveal the staining of lipid components by osmium tetroxide (Wu et al., 2011). We observed altered electron density and disorganized cuticle structure in the OE-At5g02890 and OE-Bn1 plants. These changes may have resulted from the alteration of lipid components. In summary, the cryo-SEM and TEM analyses revealed altered cuticle structure and wax crystallization in these lines, which caused the glossy phenotype.

### Chain lengths distribution of cuticular wax composition is significantly affected in OE-At5g02890 plants

**Figure 4:**
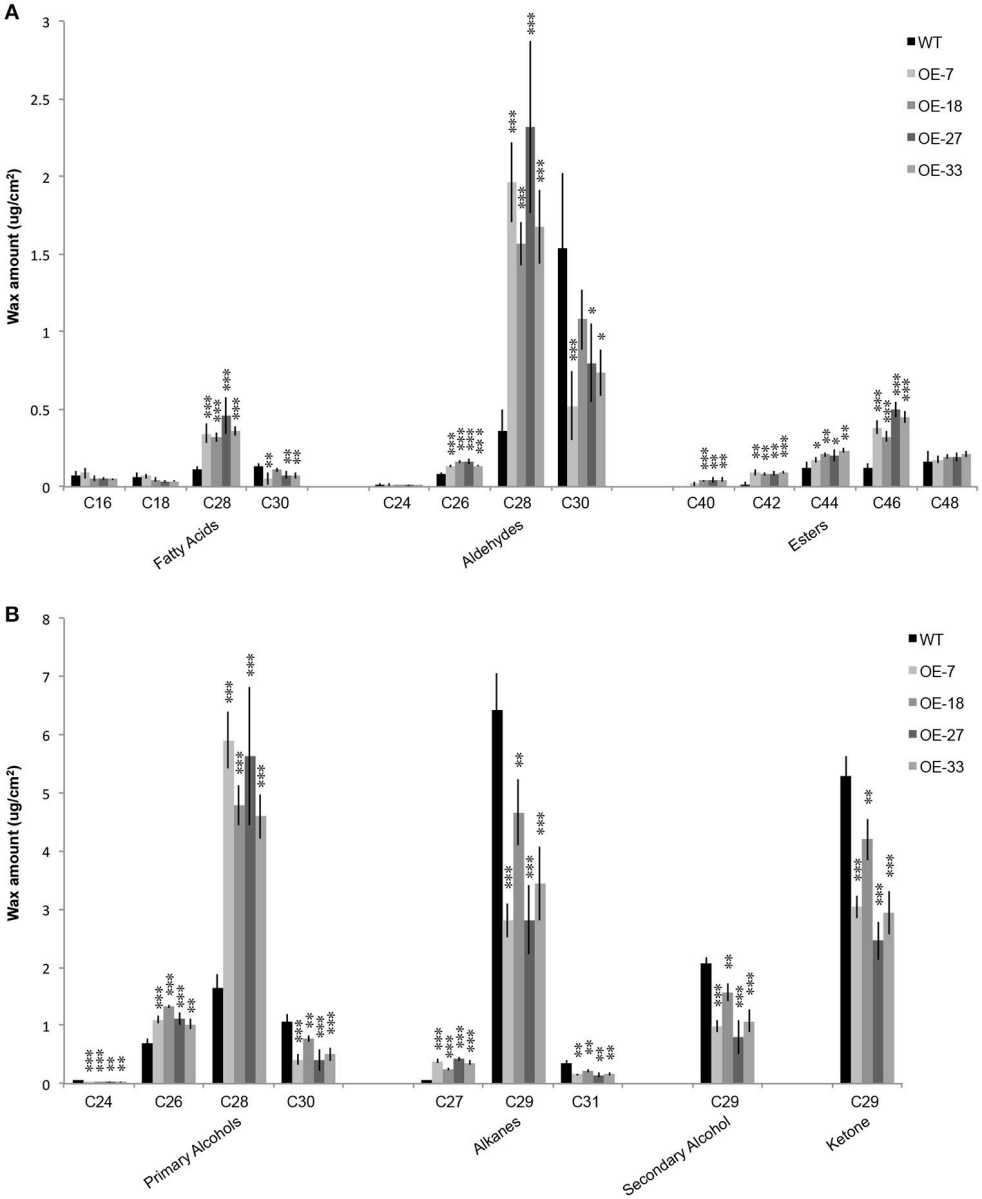
**(A,B)** Wax composition in inflorescence stems of the WT and OE-At5g02890 lines. The values represent the means of four biological replicates ± *SD* (*t*-test: ^*^p < 0.05; ^**^p < 0.01; ^***^p < 0.001). OE-7, OE-18, OE-27, and OE-33 represent different *At5g02890*-overexpressing lines.

The phenotype of the OE-At5g02890 plants suggests that At5g02890 participates in the formation of wax compounds, which mainly contain VLCFAs and their alkane, aldehyde, alcohol, ketone, and ester derivatives. To confirm this hypothesis, we monitored the cuticular wax load and composition of inflorescence stems of the four overexpressing plant lines (OE-7, OE-18, OE-27, and OE-33) and wild-type plants using gas chromatography with flame ionization detection (GC-FID) and mass spectrometry (GC-MS) analyses. Surprisingly, the total wax load did not differ between the OE-At5g02890 and wild-type plants (Figure S5). However, further analysis indicated that the chain lengths of many components were significantly altered in the overexpression plants compared with wild type. The levels of wax monomers C28 or shorter, such as C26 aldehyde, C26 primary alcohol, C27 alkane, C28 fatty acid, C28 aldehyde, and C28 primary alcohol, were higher in the overexpression plants than in wild type. By contrast, reductions were observed in the levels of all wax monomers C29 and longer, such as C29 alkane, C29 secondary alcohol, C29 ketone, C30 fatty acid, C30 aldehyde, C30 primary alcohol, and C31 alkane. Additionally, significant increases in the levels of C40, C42, C44, and C46 esters were observed in the overexpression plants (Figure 4; Figure S6A). We compared the seven major components of cuticular wax in the OE-At5g02890 and wild-type lines, finding that the OE-At5g02890 lines had significant reductions in the levels of the alkane, secondary alcohol and ketone fractions of wax (alkane-forming pathway) and significant increases in the levels of the primary alcohol and ester fractions (alcohol-forming pathway; Figure S6B). These results suggest that cuticular wax components are severely affected by the overexpression of *At5g02890*.

### At5g02890 localizes to the ER

**Figure 5:**
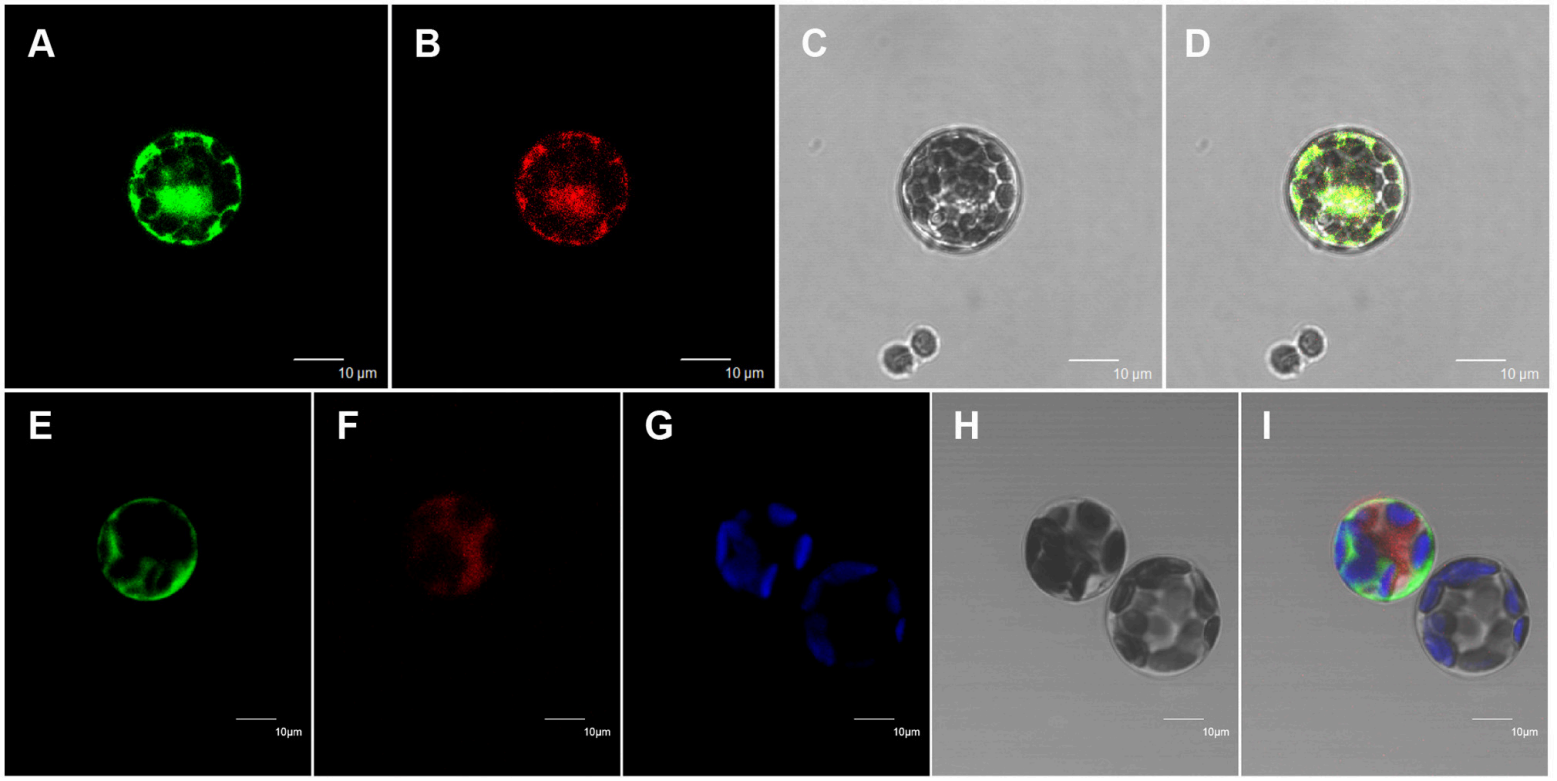
**Subcellular localization of At5g02890. (A)** Arabidopsis protoplast showing green fluorescent signals after transformation with the At5g02890-GFP and BiP-RFP fusion constructs. **(B)** The same protoplast as in **(A)** showing red fluorescent signals. **(C)** The same protoplast as in **(A)** under bright field. **(D)** Merged images from **(A–C)**. **(E)** Arabidopsis protoplast showing green fluorescent signals after transformation with the At5g02890-GFP fusion construct and an mRFP construct. **(F)** The same protoplast as in **(E)** showing red fluorescent signals. **(G)** Chlorophyll autofluorescence signals in the same protoplast as in **(E)**. **(H)** The same protoplast as in **(E)** under bright field. **(I)** Merged images from **(E–G,I)**. Scale bars = 10 μm.

All of the previously characterized wax biosynthetic enzymes are localized to the ER membrane (Samuels et al., 2008). The above results suggest that At5g02890 probably affects the synthesis of wax precursors. To investigate the subcellular localization of At5g02890, mRFP construct, and Bip:RFP fusion construct were used for cytosol marker and ER marker, respectively. The At5g02890:GFP constructs were transiently co-expressed in Arabidopsis protoplasts with the cytosol marker and ER marker via PEG/calcium-mediated transformation. As predicted, GFP and ER marker fluorescence signals overlapped, whereas the GFP and cytosol marker fluorescence signals did not overlap. The GFP signals and chlorophyll autofluorescence also did not overlap (Figure 5). This result indicates that At5g02890 is an ER-localized protein, which is consistent with its possible role in the biosynthesis of wax precursors.

### Transcriptome analysis of OE-At5g02890 plants

Overexpression of *At5g02890* alters cuticular wax composition and causes the glossy phenotype which is visible with the naked eye. First, we want to know which wax-related genes were affected; Second, we want to know whether other metabolic pathways which cause some phenotype invisible to the naked eye were affect. So we compared the transcriptional differences between OE-At5g02890 and wild-type inflorescence stems via RNA-seq using the Illumina HiSeq™ 2500 platform. To obtain reliable and comprehensive expression profiles, we evaluated six samples with three biological replicates from each experimental line. A total of 78,575,762 clean reads from six samples with an average length of 201 bp were generated. An overview of the read information is shown in Table S3. For each gene, the transcript levels were calculated and subsequently normalized to FPKM (Fragments per Kilobase of transcript per Million mapped reads), which represents gene expression levels. To identify the significantly differentially expressed genes (DEGs), we filtered all of the expressed genes using a *t*-test with restrictive conditions: *p* ≤ 0.05 and *q* ≤ 0.05. In total, 3945 DEGs were isolated from the OE-At5g02890 and wild-type inflorescence stems. Relative to the wild-type control, the OE-At5g02890 stems contained 1434 upregulated genes (Table S4) and 2511 downregulated genes (Table S5).

We performed gene ontology (GO) analysis to investigate the functions of the 3945 DEGs. All 41 groups could be categorized into three main classifications: “biological process,” “molecular function,” and “cellular component.” There were 2446 (62.02%) DEGs in the “metabolic process” category, 1742 (44.17%) in the “binding” category, and 3359 (85.17%) in the “cell” category, which were the major categories in each of the three main classifications mentioned above, respectively. In addition, large proportions of DEGs were assigned to the “cellular process,” “catalytic activity,” and “organelle” categories (Figure S7). These results suggest that *At5g02890* overexpression strongly affects metabolic activity in the plant. Furthermore, we assigned the DEGs to reference pathways in the Kyoto Encyclopedia of Genes and Genomes (KEGG) database using KeggArray software. Among the typical pathways, “metabolic pathways” (283 members) and “biosynthesis of secondary metabolites” (155 members) were the most highly represented groups. Interestingly, genes involved in “plant hormone signal transduction” (74 members) and “plant-pathogen interaction” (65 members) were enriched. In particular, 12 genes involved in “fatty acid metabolism” had significantly differential expression, which might be associated with the altered cuticular wax composition in OE-At5g02890 inflorescence stems (Figure S8). The upregulated and downregulated genes participate in diverse pathways, indicating that overexpressing *At5g02890* not only affects wax biosynthesis, but it also influences other pathways, such as plant hormone signal transduction and plant-pathogen interaction pathways.

### The expression of wax-related genes is altered in OE-At5g02890 plants

**Figure 6:**
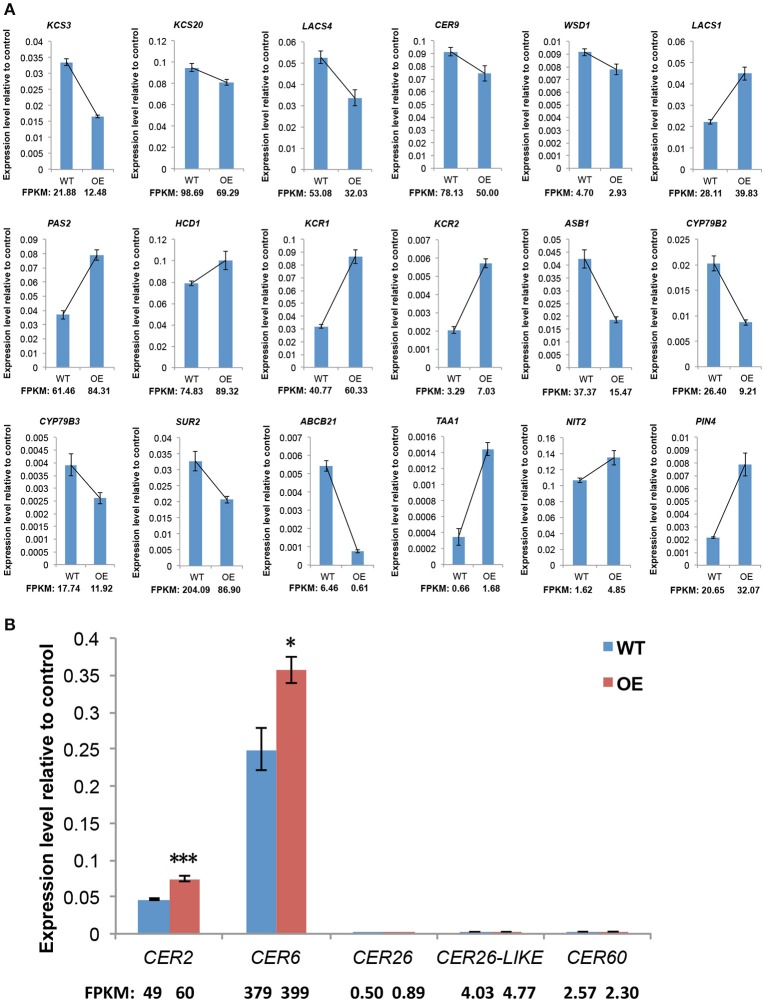
**The expression levels of genes involved in wax synthesis and auxin metabolism. (A)** qRT-PCR verification of the RNA-Seq based gene expression data. **(B)** The expression levels of genes involved in VLCFA elongation past C28. The values indicate means of four biological replicates ± *SD* (*t*-test: ^*^p < 0.05; ^***^p < 0.001). FPKM, Fragments per Kilobase of transcript per Million mapped reads. WT and OE represent wild-type lines and *At5g02890*-overexpression lines, respectively.

Cuticular wax formation is a fundamental, complex process in vascular plants that has been well described in the model plant *A. thaliana*. Therefore, we analyzed genes with identified or putative roles in cuticular wax biosynthesis, export, and regulation. Among the 74 genes, which with bold names have experimental evidences for their functions in previous work. Red arrows indicate the upregulated genes and green arrows indicate the downregulated genes (Table S6). The results show that six upregulated and five downregulated genes are involved in wax biosynthesis, whereas only one downregulated gene is related to wax export, and two upregulated genes participate in regulation of wax biosynthesis. Among the 11 cuticular wax biosynthesis-related genes, nine are involved in VLCFA elongation. Our qRT-PCR analysis of selected wax biosynthesis-related genes confirmed the high reliability of the RNA-seq data. Not surprisingly, the expression patterns revealed by qRT-PCR and RNA-Seq tended to be consistent (Figure 6A). Among these genes involved in C28 fatty acid elongation, the expression levels of *CER2* and *CER6* were significantly higher in OE-At5g02890 lines; however *CER26, CER26-LIKE*, and *CER60* show the same expression level (Figure 6B).

### Overexpression of *At5g02890* affects phytohormone homeostasis in inflorescence stems

Changes in the expression levels of groups of genes implicated in various phytohormone signal transduction pathways were identified in OE-At5g02890 inflorescence stems (Table S7). Genes involved in auxin metabolism and signaling constituted the largest group, and those involved in abscisic acid (ABA) and jasmonic acid (JA) signaling pathways were also significantly affected. We investigated identified or putative genes involved in auxin biosynthesis and transport. Auxin biosynthesis genes, such as anthranilate synthase genes (*ASA1, ASB1*), Cytochrome P450 genes (*CYP79B2, CYP79B3*), and *SUPERROOT2* (*SUR2*), showed much lower expression levels in OE-At5g02890 plants than in wild type. However, the nitrilase gene *NIT2* and *TRYPTOPHAN AMINOTRANSFERASE OF ARABIDOPSIS 1* (*TAA1*) showed enhanced transcription in OE-At5g02890 plants compared to wild type. In addition, several auxin transport and signaling translation genes were differentially expressed, showing complex expression profiles (Table S8). Similarly, we confirmed the high reliability of the RNA-seq data using qRT-PCR analysis of selected auxin metabolism-related genes (Figure 6A).

**Figure 7:**
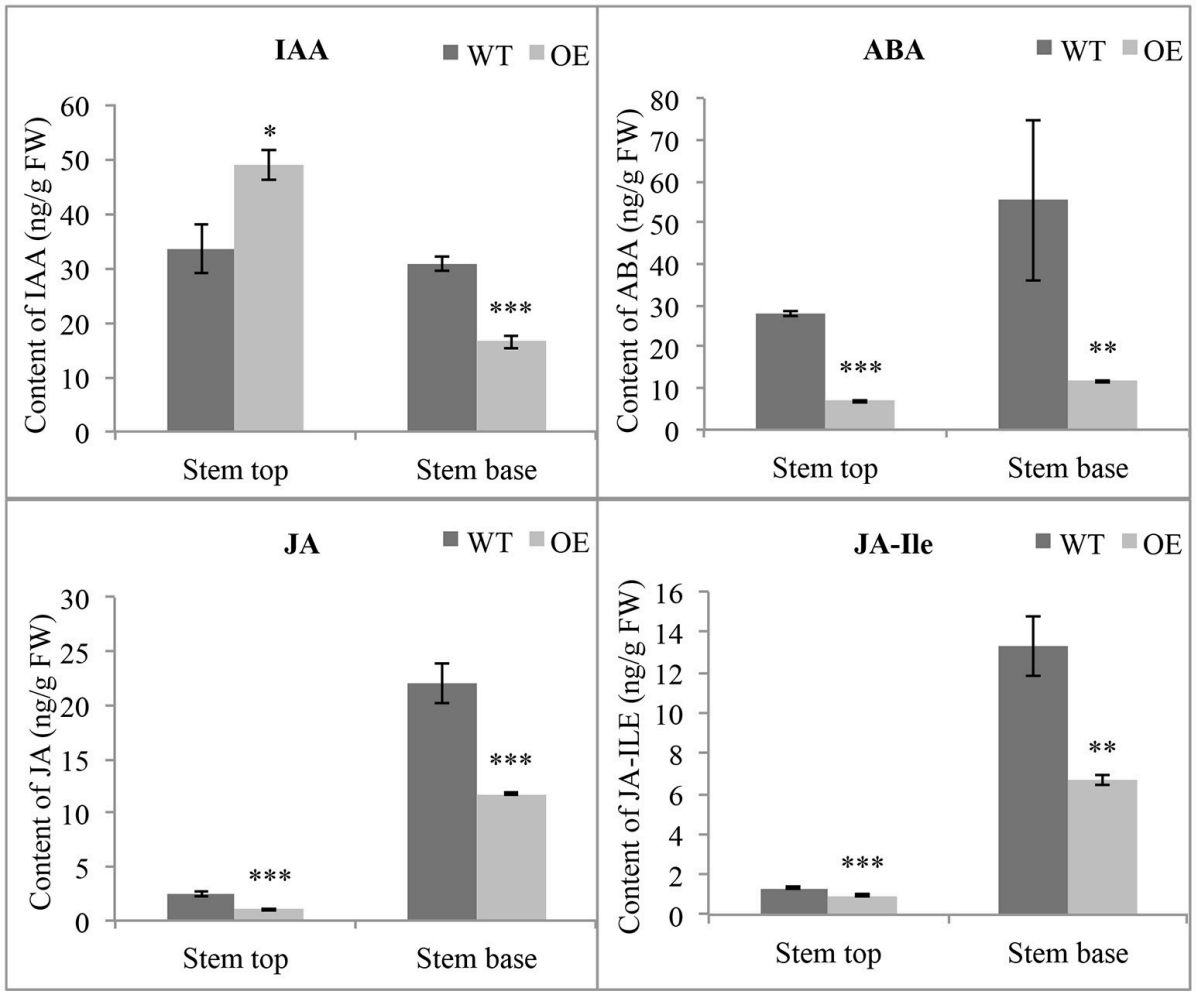
**Detection of endogenous phytohormone contents**. The endogenous IAA, ABA, JA, and JA-Ile contents in WT and OE plants were detected. The data are presented as the means ± SE from six biologically independent experiments (*t*-test: ^*^p < 0.05; ^**^p < 0.01; ^***^p < 0.001). WT and OE represent wild-type lines and *At5g02890*-overexpressing lines, respectively.

Many genes related to phytohormones have different expression levels in OE-At5g02890 plants, indicating that the phytohormone homeostasis maybe affected. So we measured the concentrations of endogenous indole-3-acetic acid (IAA), ABA, JA, and jasmonoyl-isoleucine (JA-Ile) by HPLC-MS. Obvious differences were found in the concentrations of these endogenous phytohormones between wild-type and OE-At5g02890 plants in stem tops and stem bases. The contents of endogenous ABA, JA, and JA-Ile were significantly lower in OE-At5g02890 stem tops and stem bases compared to wild type. Interestingly, the IAA content was increased by ~45% in OE-At5g02890 stem tops and reduced by ~47% in OE-At5g02890 stem bases compared to wild type (Figure 7). These results suggest that overexpressing *At5g02890* may influence ABA, JA, and JA-Ile biosynthesis and block polar auxin transport.

## Discussion

### *At5g02890* encodes a novel functional protein that is highly conserved in the *Brassicaceae*

At5g02890 is annotated as an HXXXD-type BAHD acyltransferase (http://www.arabidopsis.org). Most BAHDs contain two conserved domains: DFGWG and HXXXD. The DFGWG domain is predicted to maintain the structural stability of the enzyme but is absent or variable in some BAHDs. The HXXXD domain plays a role in catalyzing acyltransferase activity, and the histidine residue within the conserved “HXXXD” site is crucial for catalytic activity (Suzuki et al., 2003; Bayer et al., 2004; Ma et al., 2005; D'Auria, 2006; Unno et al., 2007; Yu et al., 2009; Zheng et al., 2009). Although, the characterized members of the BAHD family are associated with a broad range of metabolic pathways, most members characterized to date are soluble cytosolic enzymes (St-Pierre and De Luca, 2000; D'Auria, 2006; Panikashvili et al., 2009; Rautengarten et al., 2012). Surprisingly, At5g02890 lacks an obvious DFGWG-like motif and has an atypical HXXXD motif, which was changed to “IXXXD” (Figure S4). Our experimental results confirm that At5g02890 affects cuticular wax biosynthesis and localizes to the ER. These results indicate that the putative conserved transferase domain might not be relevant to the function of At5g02890. CER2 and CER2-LIKE1 are classified as BAHD acyltransferases based on sequence homology, but the catalytic HXXXD motif is not required for their functions (Haslam et al., 2012). In addition, At5g02890 does not contain any other previously described domains. These findings suggest that *At5g02890* probably encodes a novel functional protein with a biochemical function.

We tried to carry out loss-of-function analysis of *At5g02890* using T-DNA insertion and RNAi lines but failed to obtain such plants. Arabidopsis seeds contain C30 VLCFA derivatives, such as C29 alkane, C29 secondary alcohol, and C29 ketone (Bernard and Joubès, 2013); *At5g02890* was expressed at higher levels in seeds than in other organs, and no other paralogs of *At5g02890* were identified in *A. thaliana*; using the same vector, we only obtained five *At5g02890* RNAi plants, a number much lower than that of other genes (more than 40 RNAi plants). So we speculated that *At5g02890* mutations might be lethal which led to no T-DNA insertion and RNAi lines could be isolated. Interestingly, the homologous sequences in clade I with high levels of sequence identity to At5g02890 are all from the *Brassicaceae* (Figure S2). *A. thaliana* contains a single *At5g02890* locus, whereas some other *Brassicaceae* species contain two or more homologous genes. We therefore asked the following questions: (1) Do the homologs play similar roles to that of Arabidopsis *At5g02890*? (2) For species with multiple homologous genes, are all paralogous genes functional? To answer these questions, we investigated *B. napus*, which has more orthologous genes of *At5g02890* than any other *Brassicaceae* species. We identified four homologous genes, but only OE-Bn1 plants had glossy stems. These results indicate that the function of *At5g02890* and its orthologous genes in the *Brassicaceae* are highly conserved; however, in a particular species, the paralogous genes maybe evolve into different functions, or some of them are nonfunctional.

### Overexpression of *At5g02890* alters cuticular wax composition by affecting the extension of VLCFA from C28 to C30

The cuticular wax in Arabidopsis stems is composed of C20–C34 VLCFAs and their derivatives. In the current study, there were significantly reduced levels of cuticular wax components with more than 28 carbon atoms in OE-At5g02890 plants, whereas the levels of wax components with 28 or fewer carbon atoms were significantly increased compared to wild type (Figure 4; Figure S6A). Almost all of the stem cuticular wax components were altered, and we found that nine of 11 cuticular wax biosynthesis-related genes with different expression levels are involved in VLCFA elongation, indicating that the initial biosynthesis of VLCFAs was abnormal in these plants. Overexpressing *At5g02890* partially but significantly reduces the extension of VLCFA from C28 to C30, and the altered VLCFA composition affects the biosynthesis of the corresponding derivatives. The localization of At5g02890 to the ER is also consistent with its function in VLCFA elongation.

The levels of alkane, secondary alcohol and ketone fractions of wax were significantly reduced in the OE-At5g02890 plants due to reductions in the levels of C29 alkane, C29 secondary alcohol, and C29 ketone, the predominant members of these three groups. By contrast, the increase in levels of dominant C26 and C28 primary alcohols were responsible for the higher levels of primary alcohols. The results for wax esters are more complex, because it is generally assumed that very-long-chain alcohols are involved in the formation of alkyl esters by reacting with VLCFA precursors longer than C20 (Kunst et al., 2006). However, little is known about the preferred chain lengths of alcohol and VLCFA substrates (Lai et al., 2007). Previous studies have suggested that alcohol substrate levels, rather than ester synthase activity, limit ester biosynthesis (Lai et al., 2007). In the current study, the increased fatty alcohol levels may have resulted in higher levels of wax esters. For individual chain lengths of wax ester, the increase in C42, C44, and C46 ester levels is consistent with the altered wax esters in the wax-deficient *cer2* mutant, which also shows a significant reduction in all stem wax components longer than C28 and increased accumulation of wax components C28 or shorter compared to wild type (Lai et al., 2007; Haslam et al., 2012). In summary, overexpressing *At5g02890* had a direct or indirect negative affect on the extension of VLCFA from C28 to C30, resulting in variations in the levels of most other wax components.

### Proposed pathway explaining the probable effect of *At5g02890* on VLCFA elongation in OE-At5g02890 plants

While our results suggest that overexpression of *At5g02890* negatively affects the extension of VLCFA from C28 to C30, the exact function of At5g02890 remains elusive, particularly how it affects the extension of C28 fatty acid. We found that 11 genes associated with wax biosynthesis were upregulated or downregulated in the OE-At5g02890 lines, as revealed by RNA-Seq (Table S6). Two of these genes, *WSD1* (*At5g37300*) and *WSD1-like* (*At5g22490*), function in wax ester biosynthesis (Li et al., 2008), and the remaining nine genes are all involved in VLCFA elongation, among which the upregulated genes *KCR1, PASTICCINO2 (PAS2)*, and *CER2* are involved in C28 VLCFA elongation. We investigated the expression levels of all genes involved in VLCFA elongation past C28 in OE-At5g02890 plants by qRT-PCR. The results show that the expression levels of *CER2* and *CER6* were significantly higher in these plants than in wild type (RNA-Seq analysis showed that *CER6* was slightly but not significantly upregulated), as shown by RNA-seq, no significant differences in expression were observed for *CER26, CER26-LIKE*, and *CER60* (Figure 6B). KCR1, PAS2 and ECR/CER10 (upregulated but not significantly so) constitute the elongase complex required for VLCFA elongation in the ER with KCS enzymes which have specific substrate specificity depending on the chain length and degree of unsaturation of VLCFAs (Zheng et al., 2005; Joubès et al., 2008). *CER2* is extremely highlighted, which could promote C28 VLCFA elongation in cooperation with CER6 or CER60 (Haslam et al., 2012, 2015).

Overexpression of *At5g02890* might block VLCFA elongation from C28 to C30 via two different mechanisms. The first mechanism involves competition with substrate: *At5g02890* may be involved in other metabolic pathways that also require C28 VLCFA (For example, C28 primary alcohols, C28 aldehyde, and C27 alkane biosynthesis). However, based on previous research and our results, this hypothesis is unlikely. If this hypothesis was true, the total wax load and level of wax components C28 or shorter in OE-At5g02890 plants should be decreased as well. In addition, the expression level of *CER2* in OE-At5g02890 plants also should be reduced, but the opposite was observed (Figures 4, 6B; Figure S5). The second mechanism involves an inhibitory effect: *At5g02890* may reduce the catalytic activity of some enzymes involved in C28 VLCFA elongation. Under this scenario, in OE-At5g02890 plants, excess *At5g02890* partially blocks C28 VLCFA elongation. In order to synthesize sufficient C30 VLCFA, on one hand, additional members of the FAE complex (KCR1, PAS2, and ECR/CER10) increase the accumulation of substrates C28 and shorter VLCFAs; on the other hand, increased expression of *CER2* leads to the activation of certain enzymes (such as CER6) involved in C28 VLCFA elongation. Finally almost all the cuticular wax components are altered in these plants. However, it still remains unknown whether *At5g02890* directly affects the condensing enzymes CER6 and CER60 or other unknown proteins associated with C28 VLCFA elongation. The lack of *At5g02890* mutants leads to uncertainty that if *At5g02890* has the same function in wild plants as it in OE-At5g02890 plants. To determine the normal gene function, some approaches could be used in the future research, for example, targeted mutagenesis of *At5g02890* using CRISPR-Cas system, detecting if At5g02890 effect C28 VLCFA elongation in yeast, finding the interacting proteins of At5g02890 and testing if other wax-related proteins could interact with At5g02890.

Previous researches have shown that there are close relations between cuticular wax and phytohormones. The cuticle functions not merely as a physical barrier to minimize water loss but also mediates osmotic stress signaling and tolerance by regulating ABA biosynthesis and signaling (Wang et al., 2011). PAS1 was proposed to act as a molecular scaffold for the FAE complex in the ER, and the resulting VLCFAs are required for polar auxin transport (Roudier et al., 2010). Arabidopsis *cer9* mutant stem shows a marked lower density of wax crystals than wild-type stems and many hormone response-associated genes show altered regulation (Lü et al., 2012). Low VLCFA content increases the synthesis of the phytohormone cytokinin in the vasculature (Nobusawa et al., 2013). Our study described the alteration of cuticular wax composition and phytohormones in the same material in details. Although, the current results do not allow us to predict the biological relevance of interplay between cuticular wax and hormones, they indicate that an intimate, complex relationship exists between cuticular wax composition and plant hormone pathways, and it is an intriguing area for further research.

## Author contributions

LX carried out the experiment and data analysis and drafted the manuscript, BY provided guidance on experimental design and drafting the manuscript. VZ and LS analyzed wax composition. JG and KH analyzed transcriptome data. JW, JS, CM, JT, and TF provided help on carrying out the experiment and modification manuscript.

## Funding

This work was supported by the Natural Science Foundation of China (Grant No. 31371488, 30900904), the National High-Tech Research and Development Program of China (Grant No. 2012AA101107) and by a grant of the DFG (German Research Society) to LS.

### Conflict of interest statement

The authors declare that the research was conducted in the absence of any commercial or financial relationships that could be construed as a potential conflict of interest.
