# Supplementary material for: Overexpression of the Novel Arabidopsis Gene At5g02890 Alters Inflorescence Stem Wax Composition and Affects Phytohormone Homeostasis
Source: Front Plant Sci. 2017 Jan 26;8:68. doi: 10.3389/fpls.2017.00068 (PMC5266714; doi:10.3389/fpls.2017.00068)
Supplement: Supplementary file 1 [file Table1.DOC]

**Supplementary Table S1:** Sequences of primers used.

| **Name** | **Sequence** | **Purpose** |
| --- | --- | --- |
| AT5G02890-F | 5'-gcggagctcCCAAAATGGAAACCAAAATCC-3' | 35S-AT5G02890 construct |
| AT5G02890-R | 5'-gcgtcgacTTACTCTTTTGGCAGTGCAAC-3' | 35S-AT5G02890 construct |
| Bn1-F | 5'- gcggagctcATGGAAACCGTTGTCTCCAAATCAA-3' | 35S-Bn1 construct |
| Bn1-R | 5'- gcgtcgacTTAATGAGTAACATTTTTTGGCAAT-3' | 35S-Bn1 construct |
| Bn2-F | 5'- gcggagctcATGAAAACAATAATCCACAAGTCGAT-3' | 35S-Bn2 construct |
| Bn2-R | 5'- gcgtcgacTTAATGAGTCGCATTTTTTGGCGATG-3' | 35S-Bn2 construct |
| Bn3-F | 5'- gcggagctcATGGAAACAATAGTCCACAAGTCGAT-3' | 35S-Bn3 construct |
| Bn3-R | 5'- gcgtcgacTTAATGAGTCGCATTTTTTGGCGATG-3' | 35S-Bn3 construct |
| Bn4-F | 5'- gcggagctcATGGAAACAATAGTCCACAAGTCGAT-3' | 35S-Bn4 construct |
| Bn4-R | 5'- gcgtcgacTTAATGAGTCGCATTTTTTGGCGATG-3' | 35S-Bn4 construct |
| AT5G02890-sense-F1 | 5'-cggggtaccAAGTCAATGATCGCCGGAGTACAGACTGTC-3' | AT5G02890-RNAi construct |
| AT5G02890-sense-R1 | 5'-gcggagctcTTGGCCACTAACTCCAAACCATCATTCCCC-3' | AT5G02890-RNAi construct |
| AT5G02890-antisense-F1 | 5'-gcgtcgacTTGGCCACTAACTCCAAACCATCATTCCCC-3' | AT5G02890-RNAi construct |
| AT5G02890-antisense-R1 | 5'-cgggatccAAGTCAATGATCGCCGGAGTACAGACTGTC-3' | AT5G02890-RNAi construct |
| AT5G02890-sense-F2 | 5'-cggggtaccAAGGGTTGCGATTGCGGTCGTGATTCAGAG-3' | AT5G02890-RNAi construct |
| AT5G02890-sense-R2 | 5'-gcggagctcTTCACAATCAATACCCAACAAACATTATAAT-3' | AT5G02890-RNAi construct |
| AT5G02890-antisense-F2 | 5'-gcgtcgacTTCACAATCAATACCCAACAAACATTATAAT-3' | AT5G02890-RNAi construct |
| AT5G02890-antisense-R2 | 5'-cgggatccAAGGGTTGCGATTGCGGTCGTGATTCAGAG-3' | AT5G02890-RNAi construct |
| AT5G02890-sense-F3 | 5'-cggggtaccAAGCTGCAGCATCCTAATAGCTGATCTC-3' | AT5G02890-RNAi construct |
| AT5G02890-sense-R3 | 5'-gcggagctcTTCGTACCATCACATCCAGTACTATTTTC-3' | AT5G02890-RNAi construct |
| AT5G02890-antisense-F3 | 5'-gcgtcgacTTCGTACCATCACATCCAGTACTATTTTC-3' | AT5G02890-RNAi construct |
| AT5G02890-antisense-R3 | 5'-cgggatccAAGCTGCAGCATCCTAATAGCTGATCTC-3' | AT5G02890-RNAi construct |
| AT5G02890pro-I-F | 5'-cccaagcttCACAGCGATCTATCACCAAAGA-3' | AT5G02890-RNAi construct |
| AT5G02890pro-I-R | 5'-aactgcagTTTGGTTCGAATTGTGAAATTG-3' | AT5G02890-RNAi construct |
| AT5G02890pro-F | 5'-gcgtcgacCACAGCGATCTATCACCAAAGA-3' | Promoter coloning |
| AT5G02890pro-R | 5'-cgggatccTTTGGTTCGAATTGTGAAATTG-3' | Promoter coloning |
| AT5G02890SL-F | 5'-gctctagaATGGAAACCAAAATCCCTAAGTCAAT-3' | Subcellular localization |
| AT5G02890SL-R | 5'-gctctagaCTCTTTTGGCAGTGCAACAATAAATT-3' | Subcellular localization |
| RT-AT5G02890-F | 5'-ATTACAAAGAAGCAGGTGATAGTGG-3' | RT-PCR |
| RT-AT5G02890-R | 5'-TTAAGAAACCTGTCTCAAGGAAGAG-3' | RT-PCR |
| Actin7-F | 5'-TGGCCGATGGTGAGGATATT-3' | RT-PCR |
| Actin7-R | 5'-AACGGCCTGAATGGCAACA-3' | RT-PCR |
| qRT-AT5G02890-F | 5'-GTACTGGATGTGATGGTACGAAG-3' | qRT-PCR |
| qRT-AT5G02890-R | 5'-CAAAAGTAGATGGGAAAACAAATA-3' | qRT-PCR |
| qRT-AT5G02890-F2 | 5'-GTTACTTCATGTTGGGTTGGGTG-3' | qRT-PCR |
| qRT-AT5G02890-R2 | 5'-TTACTCTTTTGGCAGTGCAACAA-3' | qRT-PCR |
| qRT-AT5G02890-F3 | 5'-TACTTTTGGGGATGCCAAGTCG-3' | qRT-PCR |
| qRT-AT5G02890-R3 | 5'-ACAACTTCACAATCAATACCCAAC-3' | qRT-PCR |
| Actin2-F | 5'-GAAATCACAGCACTTGCACC-3' | qRT-PCR |
| Actin2-R | 5'-AAGCCTTTGATCTTGAGAGC-3' | qRT-PCR |
| qRT-KCS3-F | 5'-GCAGCGACTCGTGCTTTCATAG-3' | qRT-PCR |
| qRT-KCS3-R | 5'-CCGCTTTACCTCCAGTGTGAAT-3' | qRT-PCR |
| qRT-KCS20-F | 5'-CCAACACCGTCACTTTCTGCTAT-3' | qRT-PCR |
| qRT-KCS20-R | 5'-GCCCATACGGAAGATACAGTTAGA-3' | qRT-PCR |
| qRT-LACS1-F | 5'-GGAGCACCTTTGAGCCCTGAG-3' | qRT-PCR |
| qRT-LACS1-R | 5'-AACCGCTGGAATACCGACTGT-3' | qRT-PCR |
| qRT-LACS4-F | 5'-GGTTCTCATTGATGGATGGCTG-3' | qRT-PCR |
| qRT-LACS4-R | 5'-TCCACCGCAACATACTCTCCTT-3' | qRT-PCR |
| qRT-PAS2-F | 5'-CTGGATGGGCTCAGGTTTTGT-3' | qRT-PCR |
| qRT-PAS2-R | 5'-TGCCCCAAGTGAGAAATAGCC-3' | qRT-PCR |
| qRT-HCD1-F | 5'-CGGACTACTTCTCGGCGTTGGT-3' | qRT-PCR |
| qRT-HCD1-R | 5'-CCACCACACCTTCCGCACTATC-3' | qRT-PCR |
| qRT-KCR1-F | 5'-CAACCCACTTGGCTCCTCATTC-3' | qRT-PCR |
| qRT-KCR1-R | 5'-CTGGGCTAACTGAAAGGCGAAA-3' | qRT-PCR |
| qRT-KCR2-F | 5'-CACCACAAGATTCCTCCTCACA-3' | qRT-PCR |
| qRT-KCR2-R | 5'-TTGCTCCGTATCCTCCTTCAGA-3' | qRT-PCR |
| qRT-CER9-F | 5'-TTCACTGAGATTCCCGCCGACA-3' | qRT-PCR |
| qRT-CER9-R | 5'-TGTGCTCTGTTCTGCCTTCCTG-3' | qRT-PCR |
| qRT-WSD1-F | 5'-AAATGTGAAAGGTCCCGATGAA-3' | qRT-PCR |
| qRT-WSD1-R | 5'-TTTCCCCTTGTGTGGCAGATTT-3' | qRT-PCR |
| qRT-PIN4-F | 5'-CATTGCTTGTGGGAACTCTGTC-3' | qRT-PCR |
| qRT-PIN4-R | 5'-CTATTCCTTGAGGCAACGCAGC-3' | qRT-PCR |
| qRT-ABCB21-F | 5'-GCGGTGAAGTCCGAATAGATGG-3' | qRT-PCR |
| qRT-ABCB21-R | 5'-ATTTTCTTTCCCGTAGGCGATG-3' | qRT-PCR |
| qRT-SUR2-F | 5'-CAAGACCTCAACTTCACCGCTC-3' | qRT-PCR |
| qRT-SUR2-R | 5'-CCATACACATCTTCCTCATCTCAC-3' | qRT-PCR |
| qRT-CYP79B2-F | 5'-AAGAATCCGACATCCCAAAACT-3' | qRT-PCR |
| qRT-CYP79B2-R | 5'-ACGGTTGTGTCAGAAAGTGCCA-3' | qRT-PCR |
| qRT-CYP79B3-F | 5'-ATCACTTGACCGCTTGGCTCTAC-3' | qRT-PCR |
| qRT-CYP79B3-R | 5'-TTCCACAATAATGCCTCGTAACA-3' | qRT-PCR |
| qRT-NIT2-F | 5'-CTACAACGATACTCCCGCCACT-3' | qRT-PCR |
| qRT-NIT2-R | 5'-GATAACCACCGATAAACGCCTC-3' | qRT-PCR |
| qRT-TAA1-F | 5'-CATTGGTGAAGGACAAGGAGGT-3' | qRT-PCR |
| qRT-TAA1-R | 5'-TTGGGAAGAGTGAAAGCATCGC-3' | qRT-PCR |
| qRT-ASB1-F | 5'-TCTTCAACCCAAGTCTGGCTCC-3' | qRT-PCR |
| qRT-ASB1-R | 5'-TTGACAACAACGGAAGGTATCG-3' | qRT-PCR |
| qRT-CER2-F | 5'-GTCTACGATCACGTTCTTGGTCCTG-3' | qRT-PCR |
| qRT-CER2-R | 5'-CCCGATACCAGCTGTCCAAGTG-3' | qRT-PCR |
| qRT-CER6-F | 5'-TCTAGCTCGGTGAAGCTCAAG-3' | qRT-PCR |
| qRT-CER6-R | 5'-AAGCTCAACGGCGACAATAG-3' | qRT-PCR |
| qRT-CER26-F | 5'-CGTGGAAGGTCAATGCAATCTC-3' | qRT-PCR |
| qRT-CER26-R | 5'-TTCTCCGGTAATGGCCTTAGC-3' | qRT-PCR |
| qRT-CER26-LIKE-F | 5'-ACCAGTTGGACCAGATTTGGCA-3' | qRT-PCR |
| qRT-CER26-LIKE-R | 5'-CCCACCAGCAAAGGCTTGAGC-3' | qRT-PCR |
| qRT-CER60-F | 5'-GGTCGGATTGTATCGAACG-3' | qRT-PCR |
| qRT-CER60-R | 5'-TGCAGTTTTCTCTATTTTCCCAAT-3' | qRT-PCR |
