# Supplementary material for: Overexpression of the Novel Arabidopsis Gene At5g02890 Alters Inflorescence Stem Wax Composition and Affects Phytohormone Homeostasis
Source: Front Plant Sci. 2017 Jan 26;8:68. doi: 10.3389/fpls.2017.00068 (PMC5266714; doi:10.3389/fpls.2017.00068)
Supplement: Supplementary file 2 [file Table2.DOC]

**Supplementary Table S2.** Accession numbers of the CER27 homologs used for the phylogenetic analysis.

| **Protein** | **Species** | **Accession no.** | **Query cover** | **E value** | **Ident** |
| --- | --- | --- | --- | --- | --- |
| Uncharacterized protein | *Arabidopsis lyrata subsp. lyrata* | XP_002873068.1 | 100% | 0.0 | 88% |
| Uncharacterized protein | *Capsella rubella* | XP_006288039.1 | 100% | 0.0 | 80% |
| Uncharacterized protein | *Eutrema salsugineum* | XP_006398747.1 | 100% | 4e-180 | 73% |
| Uncharacterized protein | *Arabis alpina* | KFK24737.1 | 95% | 2e-177 | 74% |
| Uncharacterized protein | *Brassica napus* | BnaAnng00540D | 100% | 6e-173 | 71% |
| Uncharacterized protein | *Brassica napus* | BnaC02g03450D | 99% | 2e-172 | 71% |
| Uncharacterized protein | *Brassica napus* | BnaC03g00980D | 100% | 8e-172 | 70% |
| Uncharacterized protein | *Brassica napus* | BnaA03g00700D | 100% | 3e-168 | 68% |
| Uncharacterized protein | *Brassica napus* | BnaCnng03180D | 99% | 1e-163 | 71% |
| Uncharacterized protein | *Brassica napus* | BnaA10g26870D | 99% | 1e-158 | 68% |
| Uncharacterized protein | *Brassica napus* | BnaAnng00520D | 99% | 4e-149 | 68% |
| Uncharacterized protein | *Brassica rapa* | Bra028858 | 100% | 1e-127 | 67% |
| Uncharacterized protein | *Brassica oleracea* | Bol015297 | 100% | 1e-127 | 66% |
| Uncharacterized protein | *Brassica oleracea* | Bol010081 | 100% | 1e-126 | 66% |
| Uncharacterized protein | *Brassica rapa* | Bra005747 | 100% | 1e-126 | 65% |
| Uncharacterized protein | *Brassica oleracea* | Bol000771 | 97% | 1e-124 | 67% |
| Uncharacterized protein | *Brassica rapa* | Bra009559 | 97% | 1e-122 | 65% |
| Uncharacterized protein | *Brassica rapa* | Bra028859 | 100% | 1e-118 | 64% |
| Uncharacterized protein | *Jatropha curcas* | KDP39935.1 | 99% | 1e-73 | 41% |
| Putative transferase | *Ricinus communis* | XP_002531784.1 | 98% | 1e-73 | 43% |
| Uncharacterized protein | *Populus trichocarpa* | XP_006368960.1 | 98% | 5e-68 | 43% |
| Pelargonidin 3-O-(6-caffeoylglucoside) 5-O-(6-O-malonylglucoside) 4'''-malonyltransferase-like | *Citrus sinensis* | XP_006477570.1 | 98% | 9e-68 | 38% |
| Taxadien-5-alpha-ol O-acetyltransferase | *Malus domestica* | XP_008369211.1 | 98% | 1e-67 | 37% |
| Uncharacterized protein | *Citrus clementina* | XP_006440196.1 | 98% | 3e-67 | 38% |
| Putative HXXXD-type acyl-transferase family protein | *Theobroma cacao* | XP_007039716.1 | 98% | 6e-67 | 39% |
| Transferase family protein | *Populus trichocarpa* | XP_002303376.2 | 97% | 2e-66 | 41% |
| Uncharacterized protein | *Prunus persica* | XP_007210231.1 | 98% | 4e-66 | 39% |
| Uncharacterized protein | *Pyrus x bretschneideri* | XP_009378418.1 | 93% | 7e-66 | 38% |
| Anthranilate N-benzoyltransferase protein 3-like | *Pyrus x bretschneideri* | XP_009351167.1 | 98% | 1e-65 | 38% |
| Anthranilate N-benzoyltransferase protein 3 | *Prunus mume* | XP_008238389.1 | 98% | 8e-64 | 38% |
| Uncharacterized protein | *Fragaria vesca subsp. vesca* | XP_004301736.1 | 99% | 1e-62 | 38% |
| Taxadien-5-alpha-ol O-acetyltransferase | *Morus notabilis* | EXC02069.1 | 67% | 5e-58 | 44% |
| Uncharacterized protein | *Nicotiana tomentosiformis* | XP_009610046.1 | 57% | 1e-57 | 48% |
| Shikimate O-hydroxycinnamoyltransferase-like | *Nicotiana tomentosiformis* | XP_009613634.1 | 97% | 3e-56 | 36% |
| Taxadien-5-alpha-ol O-acetyltransferase | *Morus notabilis* | EXC02068.1 | 98% | 7e-55 | 36% |
