# Supplementary material for: Overexpression of the Novel Arabidopsis Gene At5g02890 Alters Inflorescence Stem Wax Composition and Affects Phytohormone Homeostasis
Source: Front Plant Sci. 2017 Jan 26;8:68. doi: 10.3389/fpls.2017.00068 (PMC5266714; doi:10.3389/fpls.2017.00068)
Supplement: Supplementary file 3 [file Table3.DOC]

**Supplementary Table S3:** Summary of RNA-seq sequencing results.

| **Samples** | **Clean reads** | **Total bases (bp)** | **Average length (bp)** |
| --- | --- | --- | --- |
| W-1 | 15850353 | 3180646752 | 201 |
| W-2 | 15221764 | 3060951001 | 201 |
| W-3 | 13836400 | 2784966892 | 201 |
| OE-1 | 9612411 | 1941333711 | 202 |
| OE-2 | 11064523 | 2234599353 | 202 |
| OE-3 | 12990311 | 2623309609 | 202 |
| Total | 78575762 | 15825807318 | 201 |

W-1, W-2 and W-3 refer to the three biological replicates of wild-type plants; OE-1, OE-2 and OE-3 refer to the three biological replicates of OE-CER27 plants.
