# Supplementary material for: Overexpression of the Novel Arabidopsis Gene At5g02890 Alters Inflorescence Stem Wax Composition and Affects Phytohormone Homeostasis
Source: Front Plant Sci. 2017 Jan 26;8:68. doi: 10.3389/fpls.2017.00068 (PMC5266714; doi:10.3389/fpls.2017.00068)
Supplement: Supplementary file 9 [file Image1.PDF]

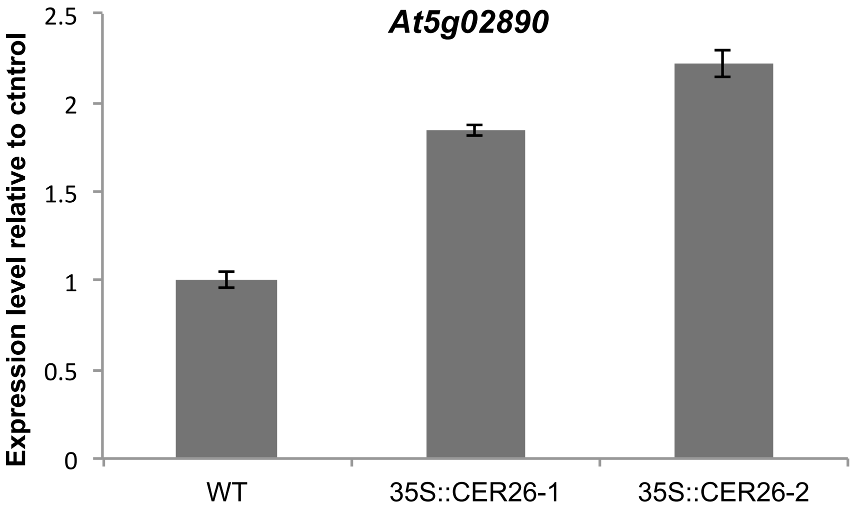

FPKM: 21.44 69.45

Figure S1. Analysis of *At5g02890* expression levels in OE-CER26 plants. qRT-PCR analysis and the RNA-Seq based gene expression of *At5g02890* in inflorescence stems of WT and OE-CER26 plants. 35S::CER26-1 and 35S::CER26-2 represent different transgenic lines. The results are presented as relative transcript abundances. The data represent the means ± SD of four biological replicates. The transcript of *At5g02890* was calculated and subsequently normalized to FPKM (Fragments per Kilobase of transcript per Million mapped reads), which represents gene expression levels.

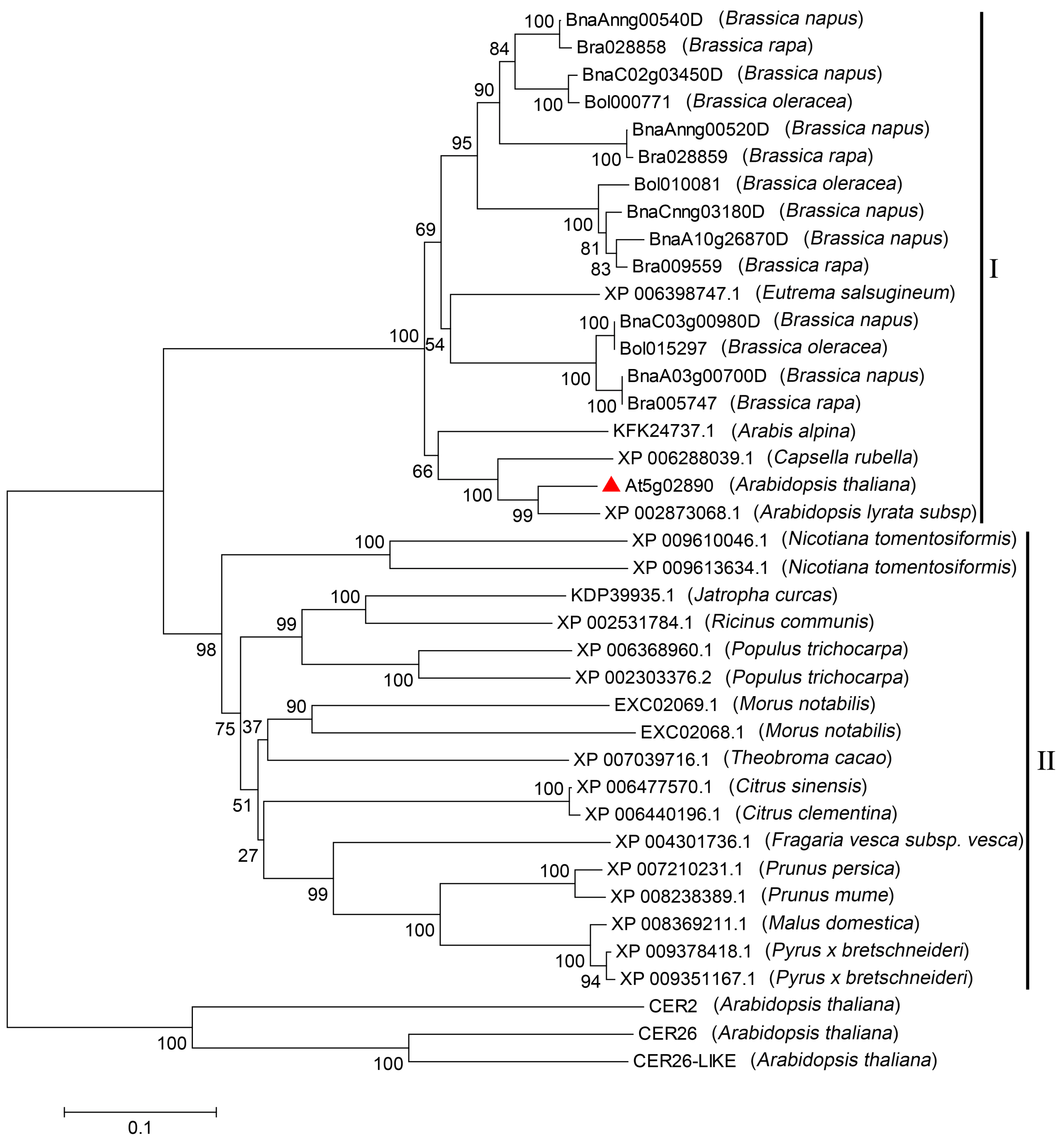

Figure S2. Unrooted phylogenetic tree of At5g02890 protein orthologs in vascular plants.

The amino acid sequences of At5g02890 orthologs were analyzed with MEGA5.2 using the neighbor-joining method. The numbers at the nodes represent percentage bootstrap values based on 1,000 replications. The length of each branch is proportional to the predicted number of amino acid substitutions per site; a scale is provided at the bottom of the tree. The red triangle indicates At5g02890.

A

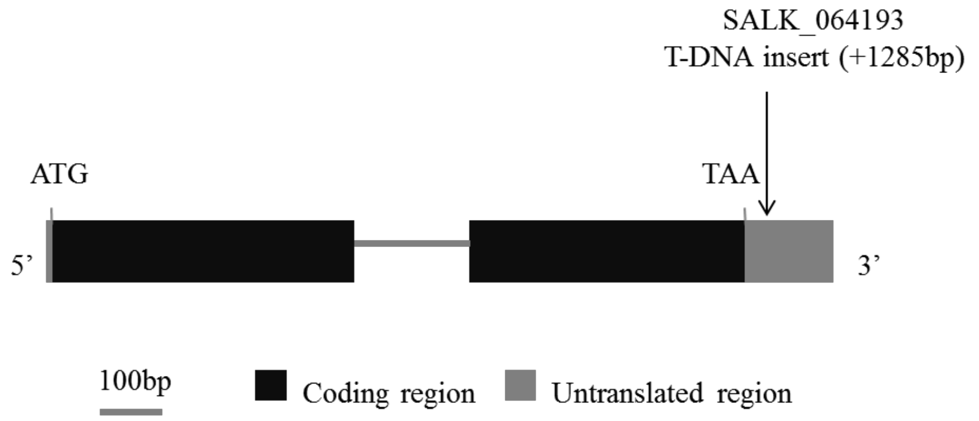
SALK_064193
T-DNA insert (+1285bp)
ATG
TAA
5'
3'
100bp
Coding region
Untranslated region

B

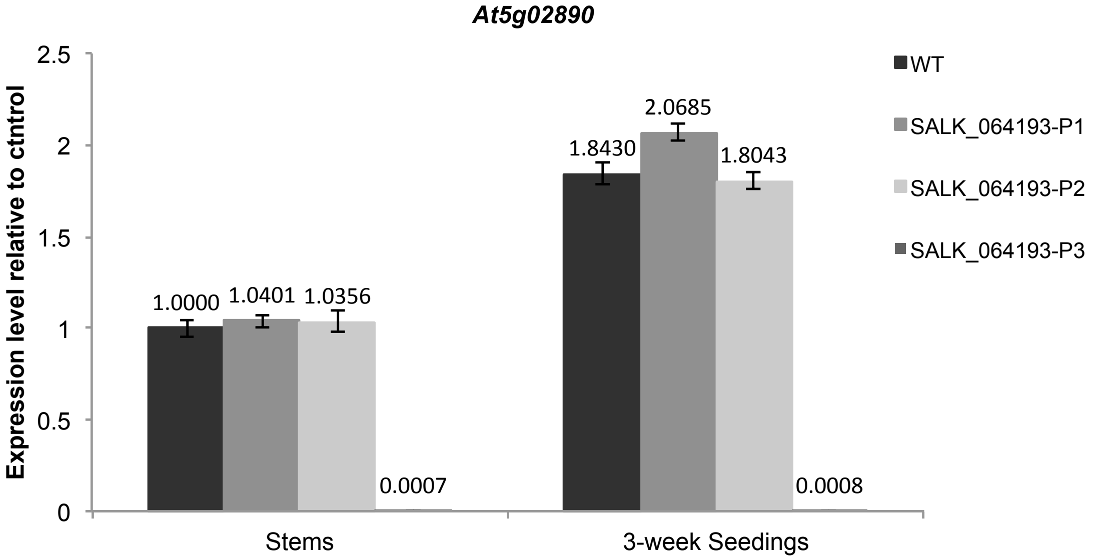
At5g02890
Expression level relative to ctntrol
2.5
2
1.5
1
0.5
0
WT
SALK_064193-P1
SALK_064193-P2
SALK_064193-P3
1.0000
1.0401
1.0356
0.0007
1.8430
2.0685
1.8043
0.0008
Stems
3-week Seedings

C

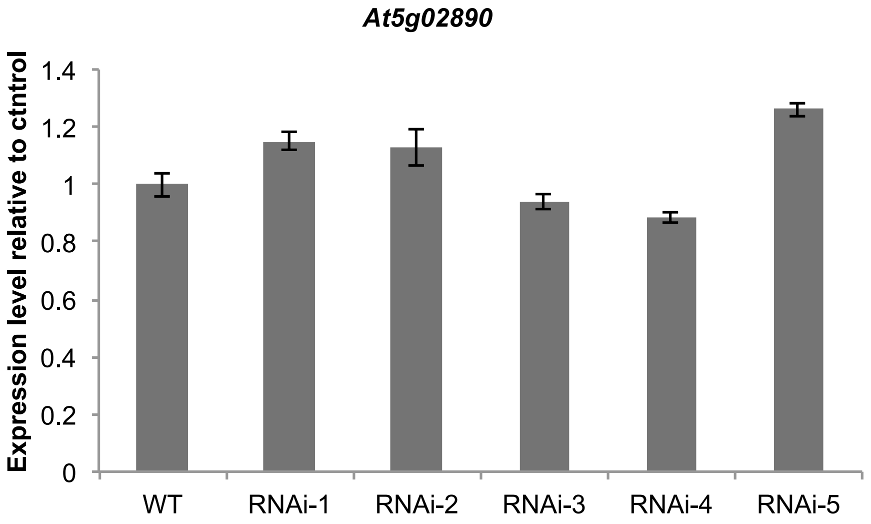
At5g02890
Expression level relative to ctntrol
1.4
1.2
1
0.8
0.6
0.4
0.2
0
WT
RNAi-1
RNAi-2
RNAi-3
RNAi-4
RNAi-5

Figure S3. Molecular characterization of the *At5g02890* mutants.

(A) Schematic of *At5g02890* gene structure. Dark boxes indicate exons, grey line indicates intron, grey boxes indicate 5'- and 3'-untranslated regions, and the arrow indicates the T-DNA insert site.

(B) qRT-PCR analysis of *At5g02890* in the T-DNA insertion plants. P1 (qRT-At5g02890-F1 and qRT-At5g02890-R1), the primers are located before the insertion site; P2 (qRT-At5g02890-F2 and qRT-At5g02890-R2), the primers are located before the insertion site and in the last 114bp of the coding sequence; P3 (qRT-At5g02890-F3 and qRT-At5g02890-R3), the primers are located after the insertion site.

(C) qRT-PCR analysis of *At5g02890* in the RNA interference plants. RNAi-1, RNAi-2, RNAi-3, RNAi-4 and RNAi-5 represent different RNA interference lines.

The results are presented as the relative transcript abundances. The data represent the means ± SD of four biological replicates.

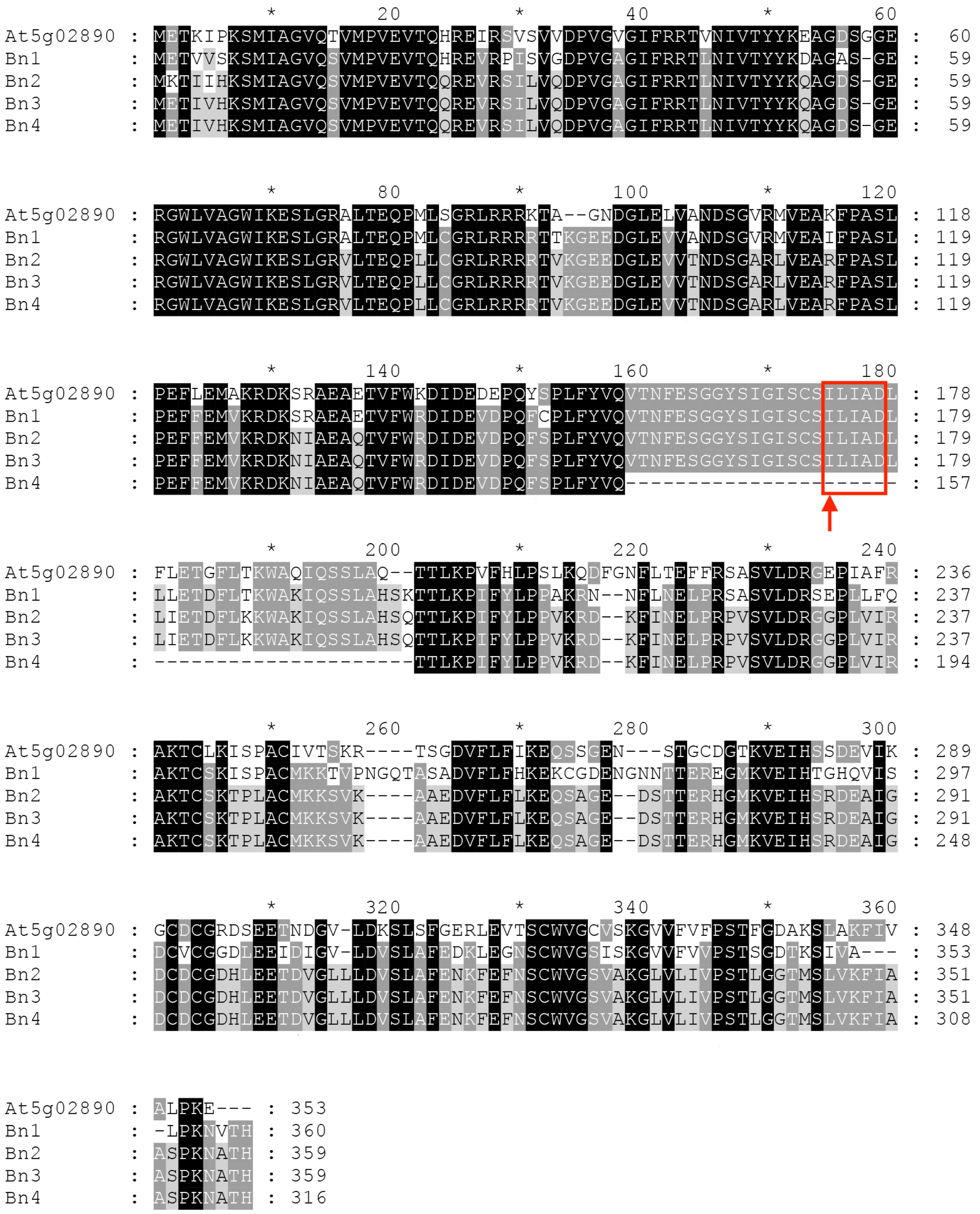

Figure S4. Amino acid sequence alignment of At5g02890 and orthologous genes from *Brassica napus*.

The predicted conserved HXXXD site is boxed, and the predicted catalytic histidine is noted with a red arrow, which indicates that the histidine is replaced by an isoleucine.

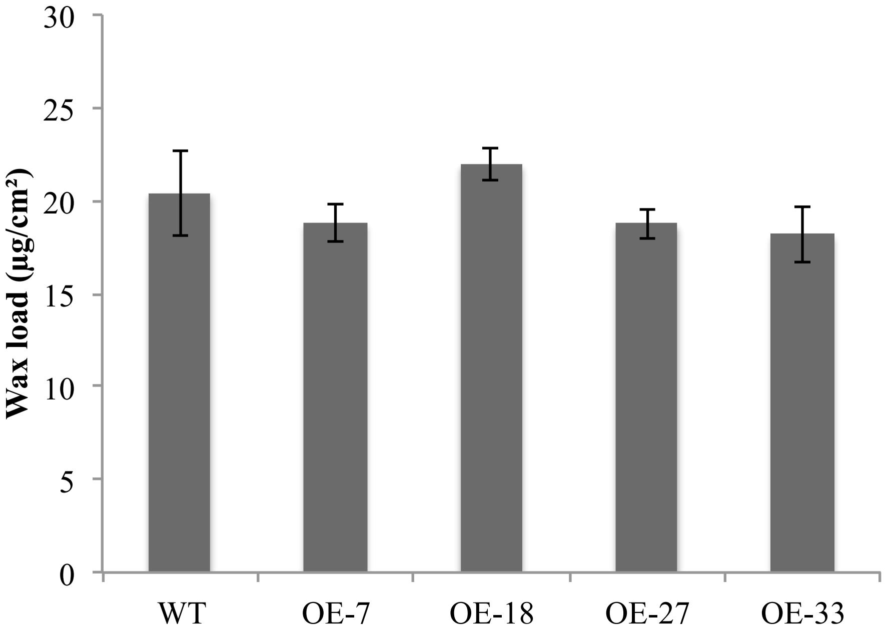

Figure S5. Total wax loads of inflorescence stems in the OE-At5g02890 lines relative to the wild-type line. The average values of four biological replicates are shown. Bars indicate the ± SD of the means. OE-7, OE-18, OE-27 and OE-33 represent different transgenic lines.

A

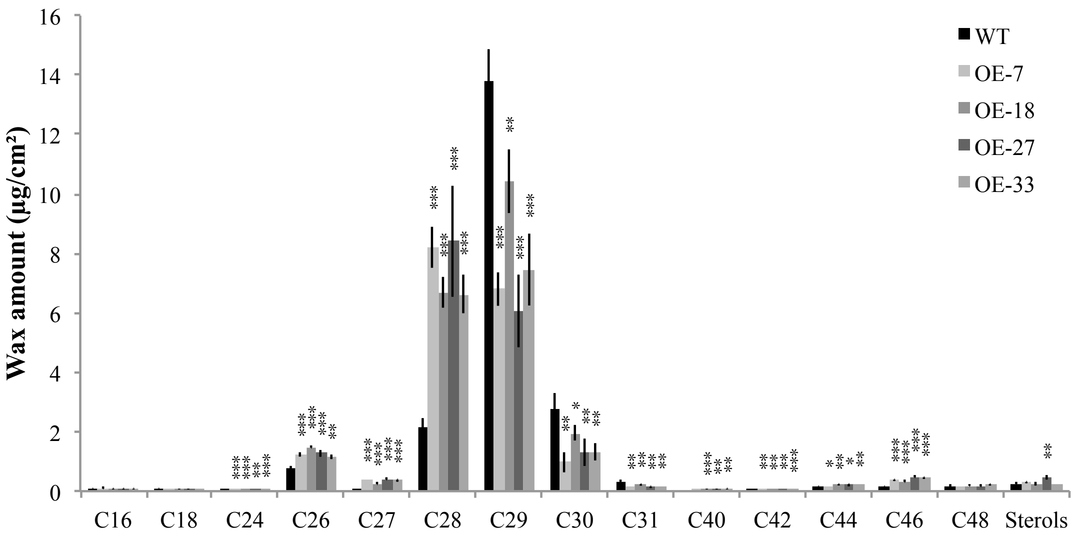

B

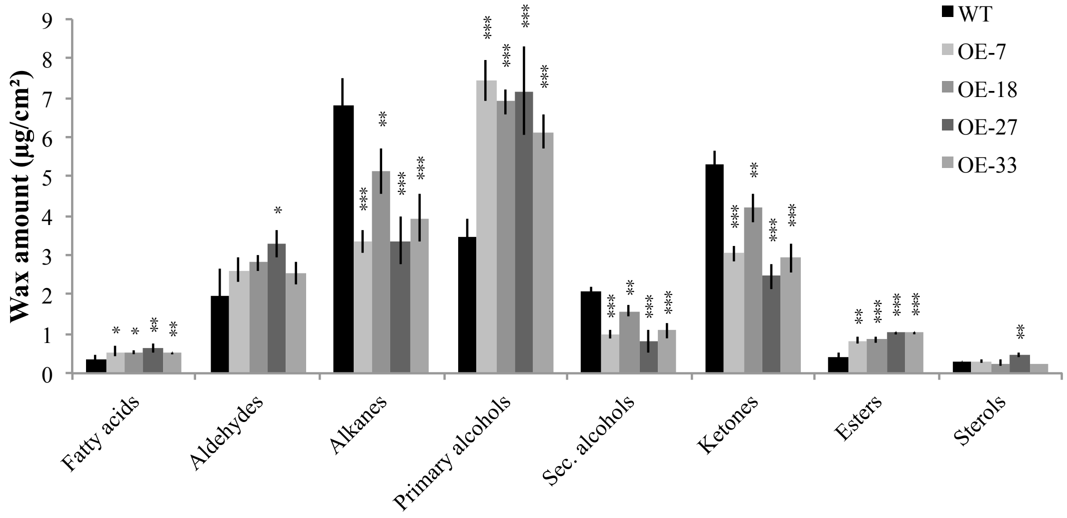

Figure S6. Comparative analysis of inflorescence stem cuticular wax components between the WT and OE-At5g02890 plants.

(A) Differential carbon chain lengths of stem cuticular wax components in the WT and OE-At5g02890 lines.

(B) Comparison of the major cuticular wax components of inflorescence stems of the WT and OE-At5g02890 lines.

The values represent the means of four biological replicates ± SD (t-test: *p<0.05; **p<0.01; ***p<0.001). OE-7, OE-18, OE-27 and OE-33 represent different transgenic lines.

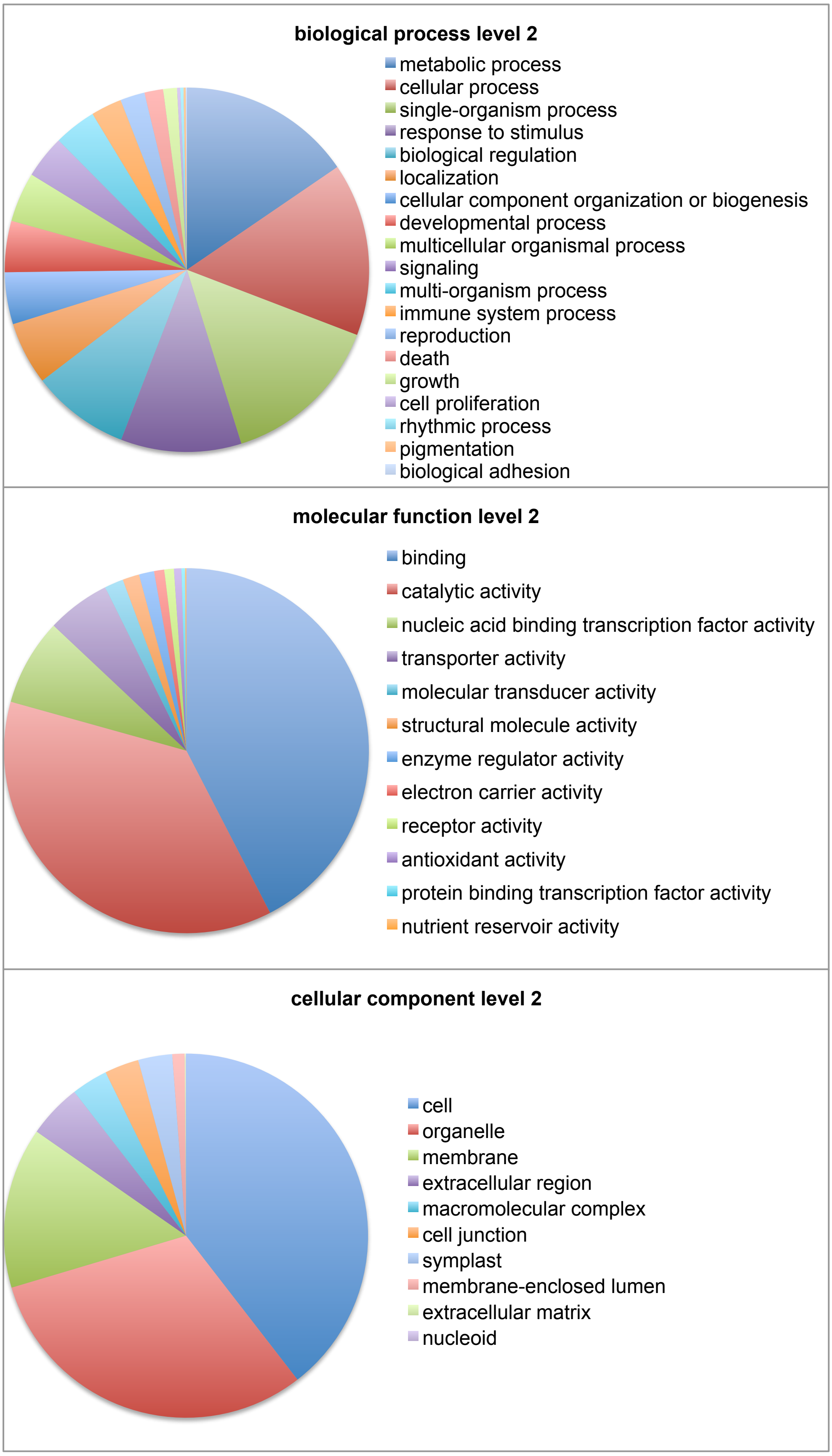

Figure S7. Classification of GO annotations.

The percentages were calculated with respect to the 3,945 DEGs.

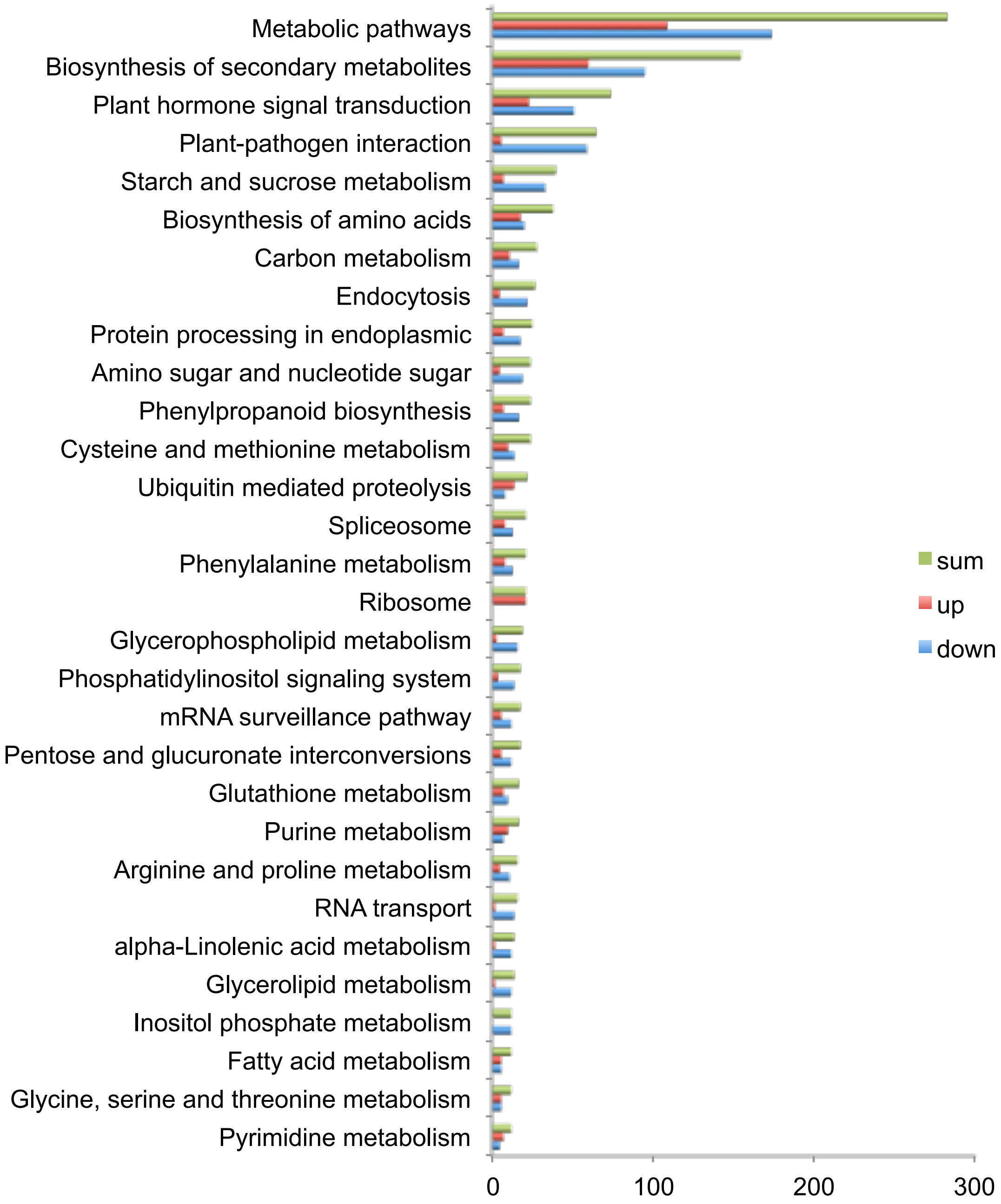

Figure S8. KEGG metabolic pathway analysis of the 3,945 DEGs.

A histogram presents the distribution of the top 30 metabolic pathways. The numbers represent the number of genes that have been annotated to each metabolic pathway.
